# Improved device efficiency and lifetime of perovskite light-emitting diodes by size-controlled polyvinylpyrrolidone-capped gold nanoparticles with dipole formation

**DOI:** 10.1038/s41598-022-05935-z

**Published:** 2022-02-10

**Authors:** Chang Min Lee, Dong Hyun Choi, Amjad Islam, Dong Hyun Kim, Tae Wook Kim, Geon-Woo Jeong, Hyun Woo Cho, Min Jae Park, Syed Hamad Ullah Shah, Hyung Ju Chae, Kyoung-Ho Kim, Muhammad Sujak, Jae Woo Lee, Donghyun Kim, Chul Hoon Kim, Hyun Jae Lee, Tae-Sung Bae, Seung Min Yu, Jong Sung Jin, Yong-Cheol Kang, Juyun Park, Myungkwan Song, Chang-Su Kim, Sung Tae Shin, Seung Yoon Ryu

**Affiliations:** 1 Division of Display and Semiconductor Physics, Display Convergence, College of Science and Technology, Korea University Sejong Campus, 2511 Sejong-ro, Sejong City, 30019 Republic of Korea; 2 Department of Applied Physics, Korea University Sejong Campus, 2511 Sejong-ro, Sejong City, 30019 Republic of Korea; 3 E-ICT-Culture-Sports Convergence Track, Korea University Sejong Campus, 2511 Sejong-ro, Sejong City, 30019 Republic of Korea; 4 Department of Physics, Chungbuk National University, Cheongju, 28644 Republic of Korea; 5 Department of Electronics and Information Engineering, Korea University, Sejong, 30019 Korea; 6 Univ. Grenoble Alpes, Univ. Savoie Mont Blanc, CNRS, Grenoble INP, IMEP-LAHC, 38000 Grenoble, France; 7 Department of Advanced Materials Chemistry, College of Science and Technology, Korea University Sejong Campus, 2511 Sejong-ro, Sejong City, 339-770 Republic of Korea; 8 Jeonju Center, Korea Basic Science Institute (KBSI), 20, Geonji-ro, Deokjin-gu, Jeonju, Jeollabuk-do 54907 Republic of Korea; 9 Busan Center, Korea Basic Science Institute (KBSI), Busan, 46742 Republic of Korea; 10 Department of Chemistry, Pukyong National University, 45 Yongso-Ro, Nam-gu, Busan, 48513 Republic of Korea; 11 Surface Technology Division, Advanced Nano-Surface Department, Korea Institute of Materials Science (KIMS), Changwon, 51508 Republic of Korea

**Keywords:** Optics and photonics, Lasers, LEDs and light sources, Inorganic LEDs

## Abstract

Herein, an unprecedented report is presented on the incorporation of size-dependent gold nanoparticles (AuNPs) with polyvinylpyrrolidone (PVP) capping into a conventional hole transport layer, poly(3,4-ethylenedioxythiophene):poly(styrenesulfonate) (PEDOT:PSS). The hole transport layer blocks ion-diffusion/migration in methylammonium-lead-bromide (MAPbBr_3_)-based perovskite light-emitting diodes (PeLEDs) as a modified interlayer. The PVP-capped 90 nm AuNP device exhibited a seven-fold increase in efficiency (1.5%) as compared to the device without AuNPs (0.22%), where the device lifetime was also improved by 17-fold. This advancement is ascribed to the far-field scattering of AuNPs, modified work function and carrier trapping/detrapping. The improvement in device lifetime is attributed to PVP-capping of AuNPs which prevents indium diffusion into the perovskite layer and surface ion migration into PEDOT:PSS through the formation of induced electric dipole. The results also indicate that using large AuNPs (> 90 nm) reduces exciton recombination because of the trapping of excess charge carriers due to the large surface area.

## Introduction

Organic–inorganic hybrid perovskites (OIHPs) are an emerging class of electronic materials that possess unique optoelectronic properties, such as high photoluminescence quantum yield, economical, high charge mobility, and high color purity. These features have made OIHP an attractive area of research in the field of organic–inorganic electronics. Perovskite light-emitting diodes (PeLEDs) have been receiving increasing attention nowadays compared to conventional organic light-emitting diodes (OLEDs) for multiple reasons. For example, top-emission OLEDs demonstrate good color purity, and the spectra show a narrow full-wave half-maximum (FWHM); however, controlling the exact device thickness for accurate micro-cavity in the commercial fabrication process of these OLED devices is difficult^1^. Additionally, quantum-dot LEDs also exhibit good color purity but they suffer from low stability^2^. Therefore, PeLEDs have attracted significant interest from researchers to mitigate these problems.

Several years ago, because of the exhibition of electroluminescence (EL) from OIHP, this material evolved as a potential candidate for PeLEDs^3^, after which many PeLEDs were fabricated using OIHP as an emissive layer; however, these PeLED devices still exhibit low efficiencies and suffer from poor stability^4–7^. One of the main reasons for the low PeLED device efficiency is the presence of a high energy barrier between the work function (WF) of poly(3,4-ethylenedioxythiophene):poly(styrenesulfonate) (PEDOT:PSS) (5.0 eV), which is a well-known hole transport layer (HTL), and the valence band of the OIHP material (5.9 eV). This barrier restricts hole injection from PEDOT:PSS to the OIHP layer, which leads to the accumulation of charges at the interface of PEDOT:PSS/OIHP, and the charge balance is disturbed in the OIHP layer. Consequently, radiative emission is quenched due to the vacancies at the grain boundary, leading to the low efficiency of PeLED^7^. To overcome such problems, multiple strategies have been employed, including guanidinium incorporation in the active layer and additive-based nanocrystal pinning, which have shown extraordinary device efficiency^8,9^. Furthermore, interfacial modification between the HTL and OIHP layer is crucial for the development of efficient PeLEDs^10–12^. However, the results of interfacial modifications on the device efficiencies remain mostly unexplained because the changes that occur in the morphology of the perovskite layer due to interfacial engineering often result in significant changes in the charge transportation/recombination of the OIHP layer. Different approaches have been adopted to improve the hole injection in perovskite devices, for example, the introduction of a composite (PEDOT:PSS/MoO_3_-ammonia) HTL, using an alternate HTL (doped CuSCN), and modification of the PEDOT:PSS layer via surfactant treatment (Triton X-100)^13–15^. In addition, the bandgap of OIHP is also controlled by changing the chemical composition such as A_α_B_1-α_X_3_ or double perovskite (A_α_B_β_C_1−α−β_X_6_)^16–19^. Although these strategies enhanced the efficiency and stability of PeLEDs to an appreciable extent, they increase the fabrication cost, and the device requires further improvements.

Metal nanoparticles (MNPs) have been incorporated to enhance the efficiency of optoelectronic devices owing to their outstanding properties. MNPs can improve the charge injection/transport properties by electrical WF alignment and optical far-field scattering^20–22^. In addition, the trap densities of MNPs affect the carrier injection barrier between the HTL and active layer. MNPs generate a strong localized surface plasmon resonance (LSPR), which maximizes the radiative recombination of charges by near-field coupling, thus increasing the external quantum efficiency (EQE) of the device^23,24^. The internal quantum efficiency (IQE) can be enhanced through magnetic-field effects of MNPs on organic emitters in OLEDs for feasible applications^25–27^. Although other methods are used for concentration or dispersion, such as inks, the size-dependent nature of MNPs gives broad dimensions to increase the efficiency of optoelectronic devices^28^. To achieve the desired results, the size of MNPs should be confined with the application of a protecting/capping material. Among the various capping materials, polyvinylpyrrolidone (PVP) is a well-known hydrophobic polymer that is used as a stabilizer, dispersant, growth modifier, and size and shape-control agent^29^. In addition, PVP helps to avoid the aggregation of MNPs because of its stable zeta potential^26^. Additionally, PVP improves the air and water stability of MNPs. In the beginning, PeLEDs showed poor EL due to the diffusion of ions from the OIHP layer to adjacent layers^8,30^. Moreover, the ion-diffusion is also responsible for the hysteresis behavior of the perovskite solar cell devices^31,32^. Furthermore, In^3+^ diffusion from indium-tin-oxide (ITO) into the active layer is one of the critical reasons for the degradation of optoelectronic devices^33^. The strong dielectric feature by PVP capping of MNPs can facilitate the prevention of ion-diffusion/migration in the OIHP layer due to the generation of an induced electric dipole to construct highly efficient and stable optoelectronics^34,35^. Therefore, a combination of HTL and PVP-capped MNPs can prove to be a useful approach to enhance the performance and stability of PeLEDs.

In the last few years, several PeLEDs have been reported using MAPbX_3_ perovskite layer. For example, Friend and co-worker reported a PeLED with a 15 nm thin CH_3_NH_3_Pb_3−x_Cl_x_ demonstrating 0.34% EQE^3^. Lee’s group constructed a green PeLEDs using CH_3_NH_3_PbBr_3_ perovskite layer and realized an EQE of 0.125%^30^. A couple of years ago, Kim et al*.* reported a PeLED using MAPbBr_3_ perovskite nano-crystal films with less surface and interfacial defects compared to solution-processed perovskite films^36^. Moreover, MNPs (Au, Ag) have also been incorporated in organic and perovskite LEDs, and perovskite solar cells to enhance the efficiency of the devices^37–40^. Herein, PVP-capped gold nanoparticles (AuNPs) of different sizes were introduced into the conventional HTL (PEDOT:PSS), and a PeLED based on a methyl-ammonium-lead-bromide (MAPbBr_3_) perovskite layer was constructed. The size-dependent properties of the AuNPs were also investigated. Moreover, PVP-capped AuNPs not only assisted the defect passivation of the OIHP layer but also improved the stability of the PeLED. A 7-fold enhancement was achieved in efficiency, with a 17-fold significant improvement in a lifetime. The results revealed that the highest efficiency and longer lifetime were demonstrated by 90 nm AuNPs compared to the other sizes of AuNPs. This advancement in device performance was attributed to the plasmonic effect of AuNPs, modified WF, carrier trapping/detrapping, and the blocking of ion-diffusion/migration by the induced dipole. To the best of our knowledge, this is the first report on the incorporation of size-controlled AuNPs with PVP capping as a modified interlayer in PeLED devices.

## Results

### PeLED device with AuNPs modified HTL and PVP passivation

**Figure 1:**
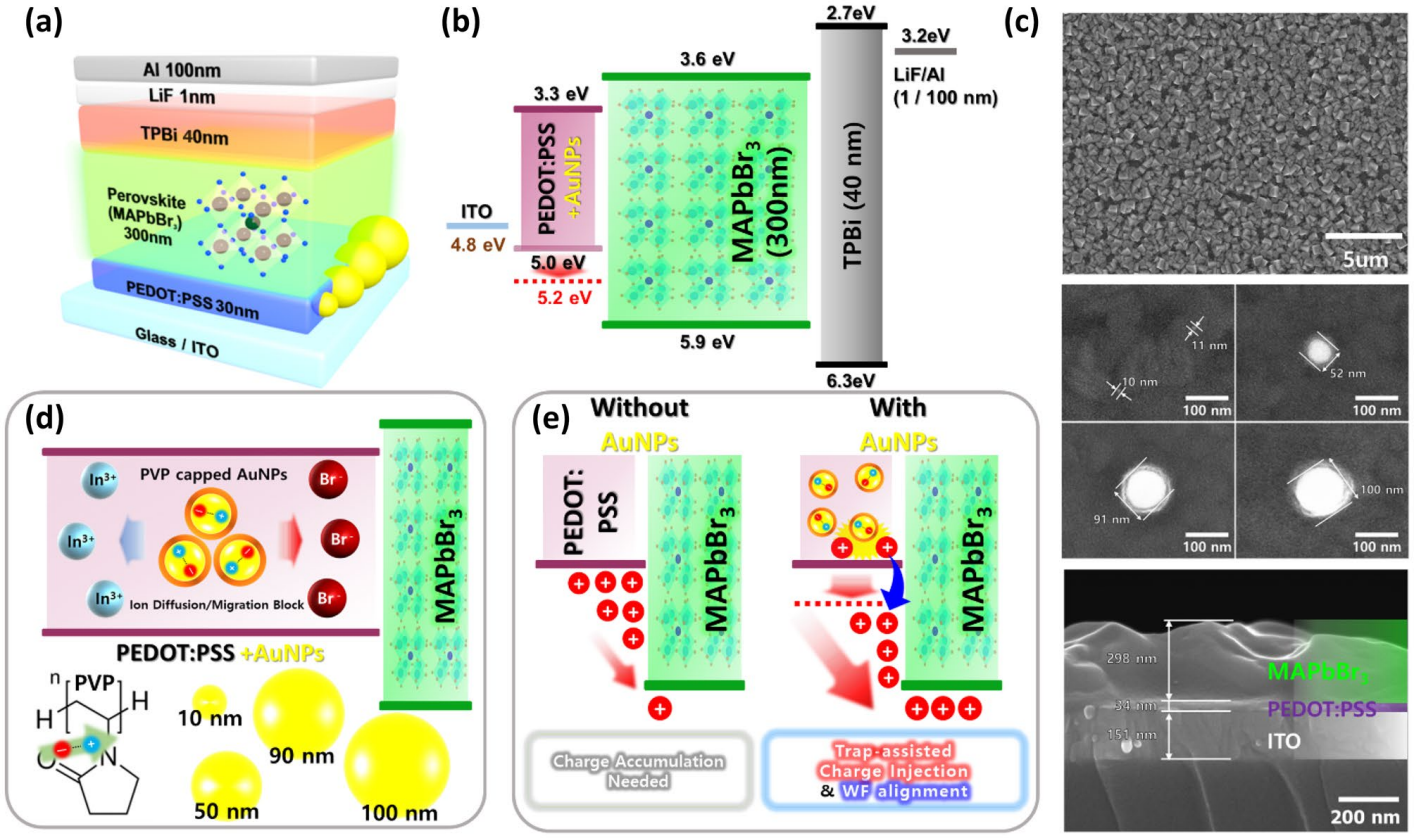
Schematic of the device structure with bandgap alignment. (**a**, **b**) Schematic of the AuNP-PeLED device structure and band alignment along with thickness of the layers. (**c**) Extended figures demonstrate the morphology of AuNPs and MAPbBr_3_ using UHR FE-SEM. (**d**, **e**) Schematic of the inside of the PEDOT:PSS with PVP-capped AuNPs, carrier trapping/detrapping, and WF alignment with and without AuNPs.

Figure 1a shows a schematic of the device structure with the component layers including PEDOT:PSS as the HTL, which was modified with AuNPs of different sizes (10–100 nm). Figure 1b displays the energy-level diagram of device, which was modulated by trap-assisted charge injection and reduction in charge accumulation at the PeLED based on AuNP-modified PEDOT:PSS. The WF of PEDOT:PSS layer was increased by incorporating AuNPs from 5.0 to 5.2 eV. It is difficult to overcome the energy gap between the PEDOT:PSS and OIHP layers in the device without AuNPs, whereas the charge carriers are injected comparably in the device containing AuNPs owing to the WF alignment (Fig. 1e). Carrier injection is decided by carrier accumulation in the device without AuNPs, while it is modified by the trapped/detrapped carriers at the AuNPs and the reduction in carrier accumulation by WF alignment at the device with AuNPs. Ultra-high-resolution field emission scanning electron microscopy (UHR FE-SEM) images of OIHP and AuNPs of different sizes, including cross-sections, are depicted in Fig. 1c. A dense and uniform OIHP (MAPbBr_3_) film was deposited on the surface of the AuNP-modified PEDOT:PSS layer. AuNPs were thoroughly embedded in the PEDOT:PSS layer and did not show any aggregation by PVP capping (UHR FE-SEM and XRD images; Supplementary Fig. S1). In addition, the XRD pattern confirmed that no significant change was observed in the samples. Furthermore, atomic force microscopy (AFM) images showed that the surface roughness of the PEDOT:PSS was increased by increasing the size of the AuNPs (Supplementary Fig. S2). Although, the surface of AuNPs is not completely covered by the PEDOT:PSS, but it was confirmed in the previous study that this strategy does not affect the device operation^41^.

The mechanism and chemical structure of the PVP-capped AuNPs are presented in Fig. 1d,e. PVP-capped AuNPs inhibited the interaction of In^3+^ ions from the ITO layer and Br^−^ ions from the perovskite layer due to the electrical dipole induced by AuNPs. Additionally, the hysteresis of *J–V* curves supports this modeling, which indicates that the formation of a dipole because the carrier transportation is affected by the Coulomb force (Supplementary Fig. S3)^42^. MNPs are often utilized in optoelectronic devices to reduce the hole injection barrier and increase the out-coupling through LSPR and scattering effects^20^. The thicknesses of PeLEDs are typically 300 nm; the direct mixing of AuNPs with enormous dimensions usually demonstrates poor device efficiency because of a high amount of leakage current due to AuNP aggregation^43^. Therefore, the capping of MNPs with any suitable material is required to avoid the leakage of current and aggregation in PeLED devices. We also employed capping materials and found PVP capping to be more suitable for AuNPs (Supplementary Fig. S4)^28^. PVP is a well-known capping material that is used as a growth modifier, stabilizer, reducing agent, and dispersing agent. The role of PVP varies depending on its synthesis procedure, and this behavior of PVP is attributed to its amphiphilic nature and molecular weight^29^. PVP-capped AuNPs inhibit the interaction of In^3+^ ions from the ITO layer and Br^−^ ions from the perovskite layer due to the induced electrical dipole by AuNPs (Fig. 1d,e). PVP-capped AuNPs exhibited a continuous change in current injection by increasing the size of AuNPs, supported by carrier trapping/detrapping from AuNPs. PVP-capped AuNPs exhibited a stable zeta potential and were well dispersed on the ITO substrate^44^. This implies that, using PVP-capped AuNPs, the defects and exciton quenching can be suppressed; hence, PeLEDs with improved stability can be realized.

A typical PeLED device structure consists of a hole injection/transport layer (HIL/HTL) interface without any electric dipole layer. In this architecture, HTL’s HOMO level is often lower than HOMO level of HIL which creates an energy barrier between HIL and HTL. Holes are transported from HIL to HTL via their HOMO level under the applied forward voltage. Due to the presence of energy barrier, some holes accumulate in this region. In our case, we have introduced PVP-capped AuNPs as an electric dipole layer to improve the hole transport. As is presented in Fig. 1d, PVP-capped AuNPs are purposed to serve as electric dipole layer, which consists of a large number of electric dipoles, is placed on the interface between PEDOT:PSS and perovskite. PVP contains a Lewis base (pyridine) that forms coordination bonds by sharing the lone pair of electrons on the N to the empty 6p orbit of Pb^2+^, and passivate, the under-coordinated Pb^2+^ vacancies. The Lewis base [carbonyl (C=O) and cyano (C=N)] efficiently passivates the traps present on the surface or at the grain boundaries. A built-in electric field is formed in each electric dipole due to the relationship between the Coulomb force and the electric field (E = F/q). Driven by an additional built-in electric field generated by the aligned electric dipoles, holes are more efficiently transported from PVP-capped AuNPs embedded PEDOT:PSS to perovskite layer, as shown in Fig. 1e.

**Figure 2:**
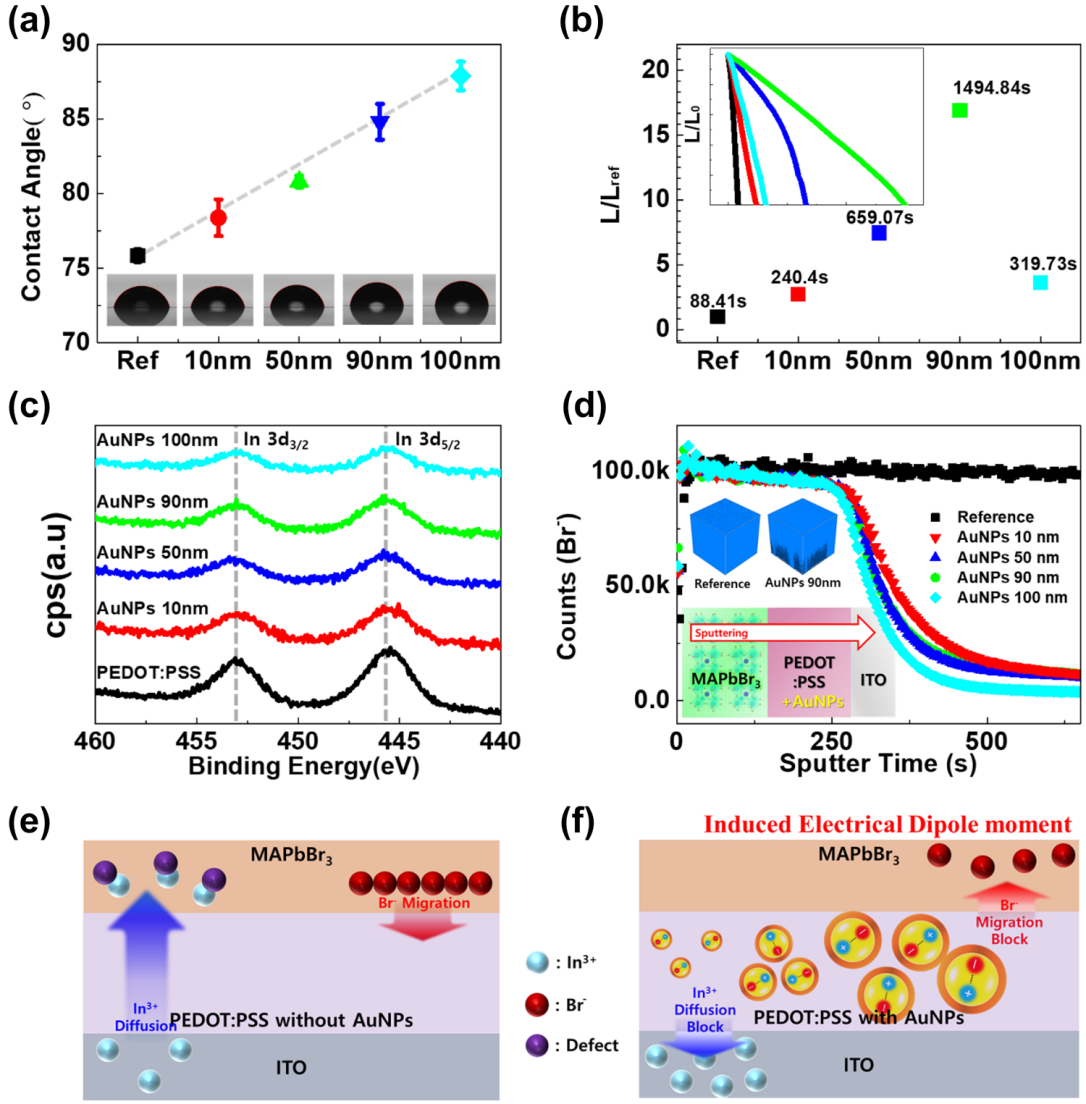
Electrical properties and surface morphology. (**a**) CA analysis with SDIW on the PEDOT:PSS and modified PEDOT:PSS. (**b**) LT50 measurement from the reference sample to the 100 nm AuNP sample, expressed as normalized based on reference. (**c**) The XPS analysis about In 3d in all the cases between PEDOT:PSS and AuNP-modified PEDOT:PSS. (**d**) The time-of-flight-secondary ion mass spectrometry (TOF–SIMS) depth profile of Br^−^ ions. The inset shows a schematic illustration of the depth profiling direction. (**e**, **f**) Schematic illustration of In penetration from ITO and surface ion migration of Br^−^ ions from the perovskite layer with/without PVP capping AuNPs.

The wetting ability of HTL surface significantly contributes to the formation of the OIHP film. A non-wetting HTL enables the migration of grain boundaries, providing a high-quality OIHP film^45^. Therefore, the water contact angle (CA) of PVP-capped AuNP-modified PEDOT:PSS was measured to evaluate the hydrophobicity of the surface. The results indicate that by increasing the size of the AuNPs, CA also increased (Fig. 2a). This behavior is ascribed to the hydrophobic nature of PVP with respect to pristine PEDOT:PSS. The maximum CA value was observed for 100-nm AuNPs (88.0°). Although the hydrophobicity was increased by PVP-capped AuNPs, the grain size of OHIP was not critically distinguishable (Supplementary Fig. S1g–i). Nevertheless, the hydrophobic nature of PVP has contributed to enhancing the stability of perovskite optoelectronics due to ion-diffusion/migration blocking^34^. Figure 2b shows that the EL lifetime data of the full device were measured at a luminance of 1000 cd m^−2^ based on a lifetime of 50% (LT50). The reference device exhibited a lifespan of 88 s, but the AuNP-modified device demonstrated high LT50 values of 240, 659, 1494, and 319 s for 10 nm to 100 nm. PeLEDs containing modified PEDOT:PSS with 90 nm AuNPs achieved a maximum LT50 value of 1494 s. According to Fig. 2b, the LT_50_ values of PeLEDs containing AuNPs were significantly enhanced by the doped HTL. This improvement appears to be due to the reduction of trap sites by the passivation layer, and the work function modified by the doped HTL improved charge carrier recombination^46,47^. Balanced hole injection in a doped HTL can minimize PL quenching at the interface, lower the operating voltage of the device, and increase brightness^47^.

In Fig. 2c, a relatively sharp In 3d peak was observed in the X-ray photoelectron spectroscopy (XPS) patterns of PEDOT:PSS, but the addition of AuNPs suppressed this peak. In addition, by increasing the size of AuNPs from 10 to 100 nm, the peak was further suppressed, which is beneficial for device lifetime and stability. Supplementary Fig. 5 shows the WF of the AuNP-modified PEDOT:PSS film. Modified PEDOT:PSS with 100 nm AuNPs demonstrated a superior WF (Φ = 5.2 eV) as compared to pristine PEDOT:PSS (Φ = 5.0 eV)^48,49^, therefore, it was anticipated that a smooth and better hole injection will take place from ITO to OIHP^28^. Additionally, time-of-flight secondary ion mass spectrometry (TOF–SIMS) analysis was performed to confirm the distribution of Br^−^ ions from perovskite to ITO with and without AuNPs, as shown in Fig. 2d and Supplementary Fig. S6. In the reference sample, Br^−^ ions were maintained throughout the region, but Br^−^ ions rapidly decreased in the samples in which AuNPs were inserted into PEDOT:PSS. As shown in Fig. 2d, the Br^−^ ions exist at high concentrations until a sputtering time of 250 s at the interface between OIHP and PEDOT:PSS. However, after 250 s, the concentration of Br^−^ ions decreased rapidly compared to the reference sample. The depth profiles of the isotopic form of Br^−^ combined with O showed the behavior of the Br^−^ ions. The results of the distribution of ^81^Br^−^, ^81^BrO^−^, ^81^BrBr^−^, and ^81^Br_2_^−^ show that Br^−^ ions are pushed back^50^. Moreover, the 3D distribution image of Br^−^ ions indicates that Br^−^ ions are completely diffused in the reference sample. There will be a difference before and after the operation, but the obtained results were not clearly distinguishable (data not shown). However, in the presence of AuNPs, an empty area at the bottom was observed, which indicates that the migration of Br^−^ ions is blocked, as shown in the inset of Fig. 2d and Fig. S6e. Thus, we can conclude that the size of the PVP-capped AuNPs affects the kinetics of Br^−^ ion blocking. In summary, in the absence of AuNPs (Fig. 2e), In^3+^ ions penetrate and react with the Br^−^ ions on the surface of perovskite to create defects, negatively affecting the lifetime of the device^51,52^. However, in the presence of PVP-capped AuNPs (Fig. 2f), ion migration is suppressed, and thus, the lifetime of the device is improved^34,53^. This may be attributed to the electrically induced dipole moment of PVP-capped AuNPs, which was confirmed by the hysteresis of the *J–V* curves, supporting this modeling (Supplementary Fig. S3)^42^.

### Device performance with and without AuNPs

**Figure 3:**
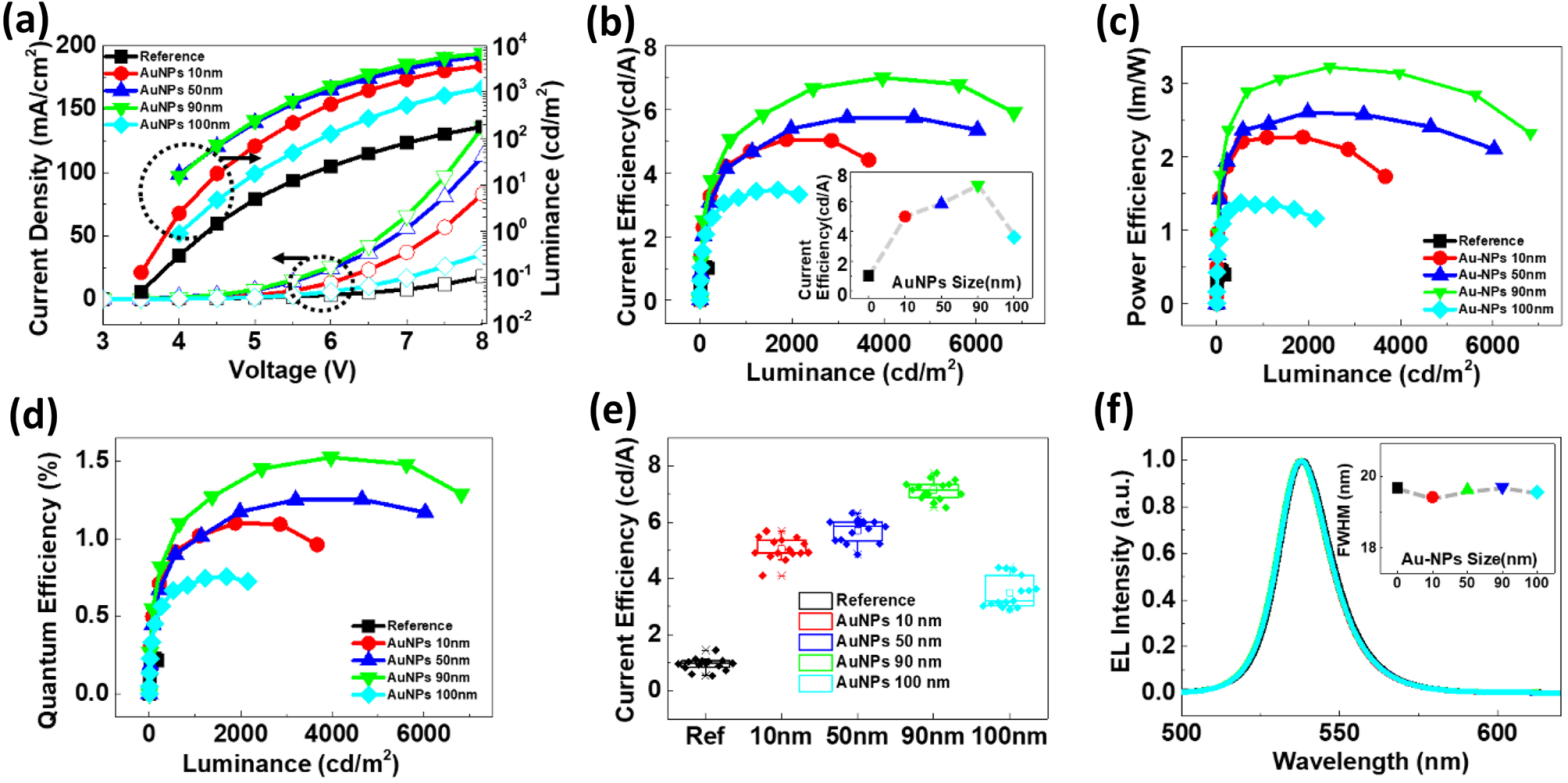
Device performance of the PeLED devices with and without AuNPs. (**a**) Current density–voltage–luminance (**b**) Current efficiency (inset: the trend of current efficiency depending on AuNPs (**c**) Power efficiency–luminance (**d**) EQE–luminance plots. (**e**) The statistical data of current efficiency (**f**) EL spectra of green PeLEDs with AuNP-modified PEDOT:PSS.

The device performances of all fabricated PeLEDs with various PVP-capped AuNPs (10–100 nm) are displayed in Fig. 3. The reference device without AuNPs showed the lowest current and luminance in the current density and luminance versus voltage (*J–V–L*) characteristics. Figure 3a presents the *J–V–L* characteristics of all PeLEDs containing AuNPs of different sizes. All PeLEDs containing AuNPs exhibited higher current density and luminance than the reference device by trap-assisted charge injection and reduction of carrier accumulation, which enhanced the carrier injection. Among the different sizes of AuNPs, PeLEDs containing 90 nm AuNPs achieved the highest current density and luminance. By contrast, PeLEDs having 100 nm AuNPs showed the lowest current density and luminance. A similar trend was obtained for the current efficiency versus luminance (*CE–L*), power efficiency versus luminance (*PE–L*), and EQE versus luminance (*EQE–L*) characteristics (Fig. 3b–d). At 1000 cd m^−2^ luminance, the 90 nm AuNP-modified PEDOT:PSS-based PeLED showed a CE of 5.45 cd A^−1^, PE of 2.96 lm W^−1^, and an EQE of 1.23%. PeLEDs with 90 nm AuNP-modified PEDOT:PSS demonstrated the best device performance with a maximum CE of 7.12 cd A^−1^, PE of 3.23 lm W^−1^, and an EQE of 1.53% (Table 1). The low device performance of PeLEDs using larger (100 nm) AuNP-modified PEDOT:PSS can be ascribed to the poor charge balance due to the higher carrier trapping by the larger AuNP surface, which degraded the current injection. In addition, when the density of MNPs was too high, or when the distance between the excitons and MNPs was too small, non-radiative decay of excitons occurred on the surface of MNPs^43^. Although it is generally lower than the currently reported device efficiency, we wanted to see the effect of AuNPs-modified PEDOT:PSS on the basic structure. To ensure reliability and reproducibility, the statistical data for each size of AuNPs, including the reference sample, are displayed in Fig. 3e. Here, we confirmed the reproducibility and found the same trend as observed in Fig. 3b–d. The EL spectra of all devices were almost the same, showing a green emission at 538 nm (Fig. 3f). This peak at 538 nm is ascribed to the characteristic peak of MAPbBr_3_^8,54^. No critical shift was observed in the EL spectra of the devices, which indicates that the variation in the size of AuNPs ≤ 100 nm does not affect the EL and FWHM behavior. These results revealed that AuNPs enhanced the optical properties of green PeLEDs, whereas the color shift was not critically induced, and angle-dependence is negligible, as displayed in Supplementary Fig. S7.

**Table 1:** Device performance (luminance, CE, PE, and EQE) of the PeLED devices with/without AuNPs. Color coordinates are presented in compliance with the International Commission on Illumination (CIE-1931).

	Luminance (cd m^−2^)	Current efficiency (cd A^−1^)	Quantum efficiency (%)	Power efficiency (lm W^−1^)	Color coordinate (x, y)	FWHM (nm)
Reference	214	1.00	0.22	0.38	(0.2438, 0.7321)	19.68
AuNPs 10 nm	1906	5.03	1.08	2.25	(0.2455, 0.7325)	19.42
AuNPs 50 nm	3204	5.87	1.26	2.61	(0.2483, 0.7308)	19.61
AuNPs 90 nm	3922	7.12	1.53	3.23	(0.2471, 0.7318)	19.71
AuNPs 100 nm	1676	3.60	0.75	1.32	(0.2483, 0.7309)	19.56

### TRPL measurement and simulation for optical enhancement

**Figure 4:**
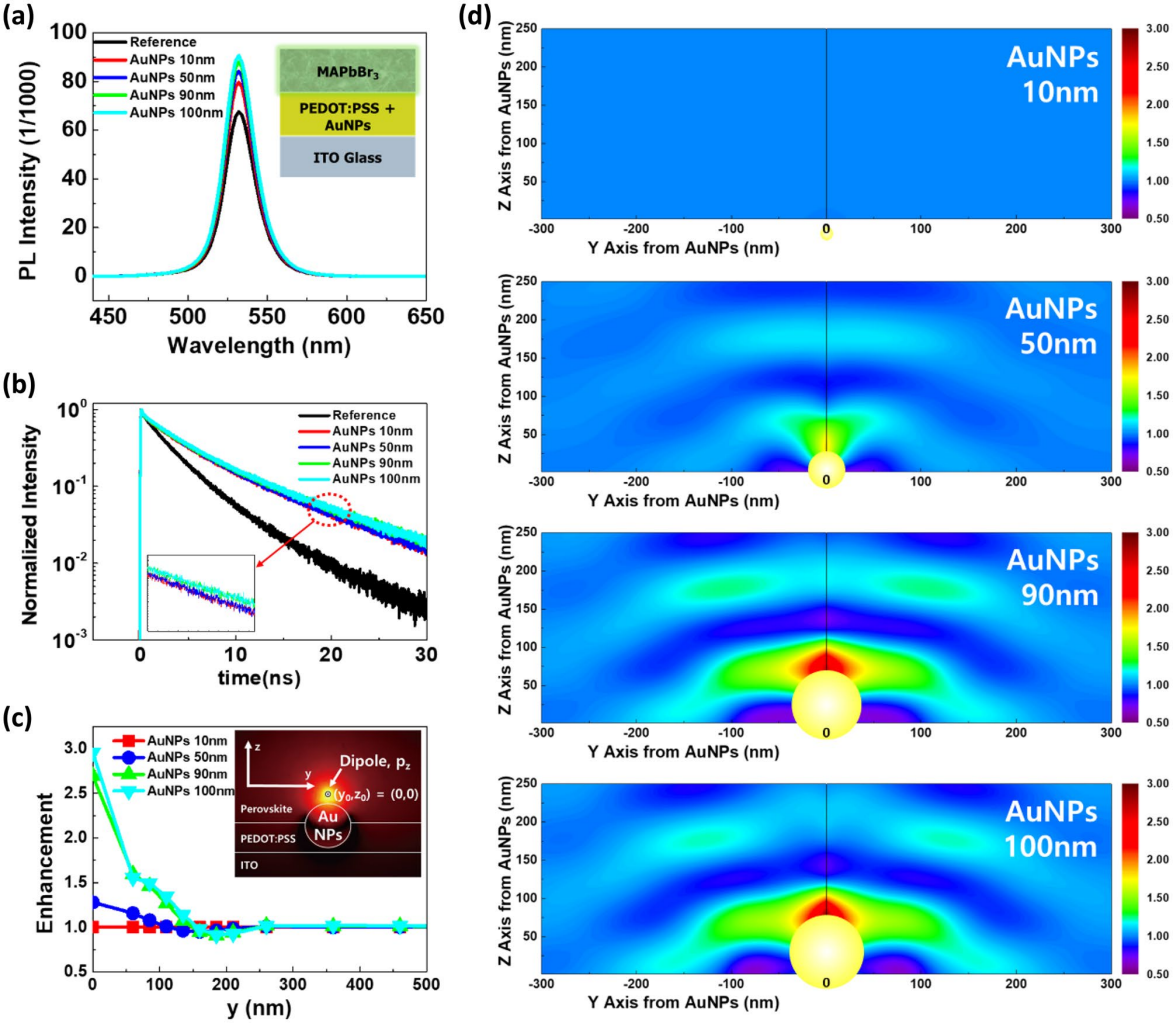
PL and TRPL spectra and optical simulation analysis of AuNP-PeLED. (**a**, **b**) Steady-state PL spectra and TRPL result based on the sizes of AuNPs. (**c**, **d**) Optical simulation data for the enhancement of out-coupling by AuNPs with different diameters.

The improved device performance with AuNPs is not only due to electrical modeling but also because of the effective optical plasmonic interactions with excitons generated in the OIHP layer. To clearly understand the mechanism of interaction between the AuNP plasmons and perovskite excitons, we recorded the photoluminescence (PL) and time-resolved photoluminescence (TRPL) for the MAPbBr_3_ perovskite layer with and without AuNPs in PEDOT:PSS, as depicted in Fig. 4a,b. The PL and TRPL results show that the lifetime of excitons was increased by inserting AuNPs, which have different absorbance peaks (Supplementary Fig. S8). Although the absorbance of AuNPs was red-shifted as the size of AuNPs increased^55^, the PL spectra were not seriously affected, revealing that the enhancement is mainly based on the far-field effect, rather than the LSPR (near-field effect). The PL intensity (expressed in 1/1000) indicates that the number of absolute photons increased with the increasing size of AuNPs, resulting in enhanced out-coupling. Moreover, the TRPL results also revealed that the lifetime of excitons was enhanced by increasing the size of the AuNPs (Table 2). This implies that the increased number of emitted photons resulted from the far-field scattering effect by AuNPs^56^. This implies that the loss of photons was reduced due to the scattering effects of AuNPs, which is in agreement with the steady PL spectra and device performance. Our perovskite films emitted a green light at 532 nm and the intensity trend was 100 nm > 90 nm > 50 nm > 10 nm > pristine. The largest increment in the intensity was observed for the 100 nm AuNPs sample. Steady state PL spectra show an increase in out-coupled light quantitatively by increasing the size of AuNPs, and the PL quenching is reduced. Our PL results confirm that the luminescence efficiency can be enhanced by inserting a PVP-capped AuNPs at the interface with perovskite layer. TRPL spectra show an increase in excitons lifetime due to scattering of light. By fitting the PL decay curves with a tri-exponential decay model^57^ (Fig. 4b) three decay pathways were received, which can be called as; τ_1_ (fast), τ_2_ (middle), and τ_3_ (slow). The τ_3_ (slow) pathway was associated with the “radiative recombination within the grains”, whereas the τ_1_ (fast) and τ_2_ (middle) pathways were ascribed to “two types of trap-assisted recombination at grain boundaries”. Since the only change we made in the overall device structure is adding AuNPs to PEDOT:PSS, PL and TRPL spectra can provide a direct indication of the PL amplification of perovskite film accelerated excitons decay in the presence of AuNPs.

**Table 2:** Parameters of the TRPL spectroscopy based on the AuNPs modified PeLEDs (reference, AuNPs 10 nm, AuNPs 50 nm, AuNPs 90 nm, and AuNPs 100 nm, respectively).

	A_1_ (%)	τ_1_ (ns)	A_2_ (%)	τ_2_ (ns)	A_3_ (%)	τ_3_ (ns)
Reference	24.0	0.109	54.4	2.33	21.6	5.9
AuNPs 10 nm	24.9	0.141	41.4	3.12	33.7	9.07
AuNPs 50 nm	21.7	0.141	44.6	3.24	33.7	9.19
AuNPs 90 nm	22.9	0.152	43.6	3.32	33.5	9.89
AuNPs 100 nm	20.2	0.168	46.3	3.41	33.5	9.96

Furthermore, full-wave optical simulations were performed to confirm the optical amplification by the AuNPs. Figure 4c shows the radiation enhancement of a dipole near the AuNPs as a function of the dipole position relative to the AuNPs on the Y-axis (within 200 nm), with varying diameters of AuNPs. For 10-nm AuNPs, radiation enhancement was negligible owing to the small surface and scattering area; however, the optical amplification was significantly improved when AuNPs with large diameters were applied. Figure 4d shows the map for optical amplification with different dipole locations relative to AuNPs on the Y- and Z-axes, as displayed in the inset of Fig. 4c. The optical amplification was negligible for all dipole locations with the 10-nm AuNPs (Fig. 4c). For 50-nm AuNPs, the optical amplification was slightly improved near the AuNPs (y = 0 nm and z = 0 nm). By contrast, a large optical amplification was observed near AuNPs of 90 and 100 nm. These results indicate that carefully chosen AuNPs with optimized sizes, such as 90 and 100 nm, improve the performance of PeLEDs to a greater extent because of the strong optical amplification near AuNPs. However, the device performance of the 100-nm AuNPs was severely degraded. Therefore, not only the optical plasmonic effect but also the electrical modeling (such as trap density, carrier accumulation, and injection barrier) should be investigated to describe the device efficiency trend.

### Electrical property analysis (cole–cole plot and trap-filled limit density)

**Figure 5:**
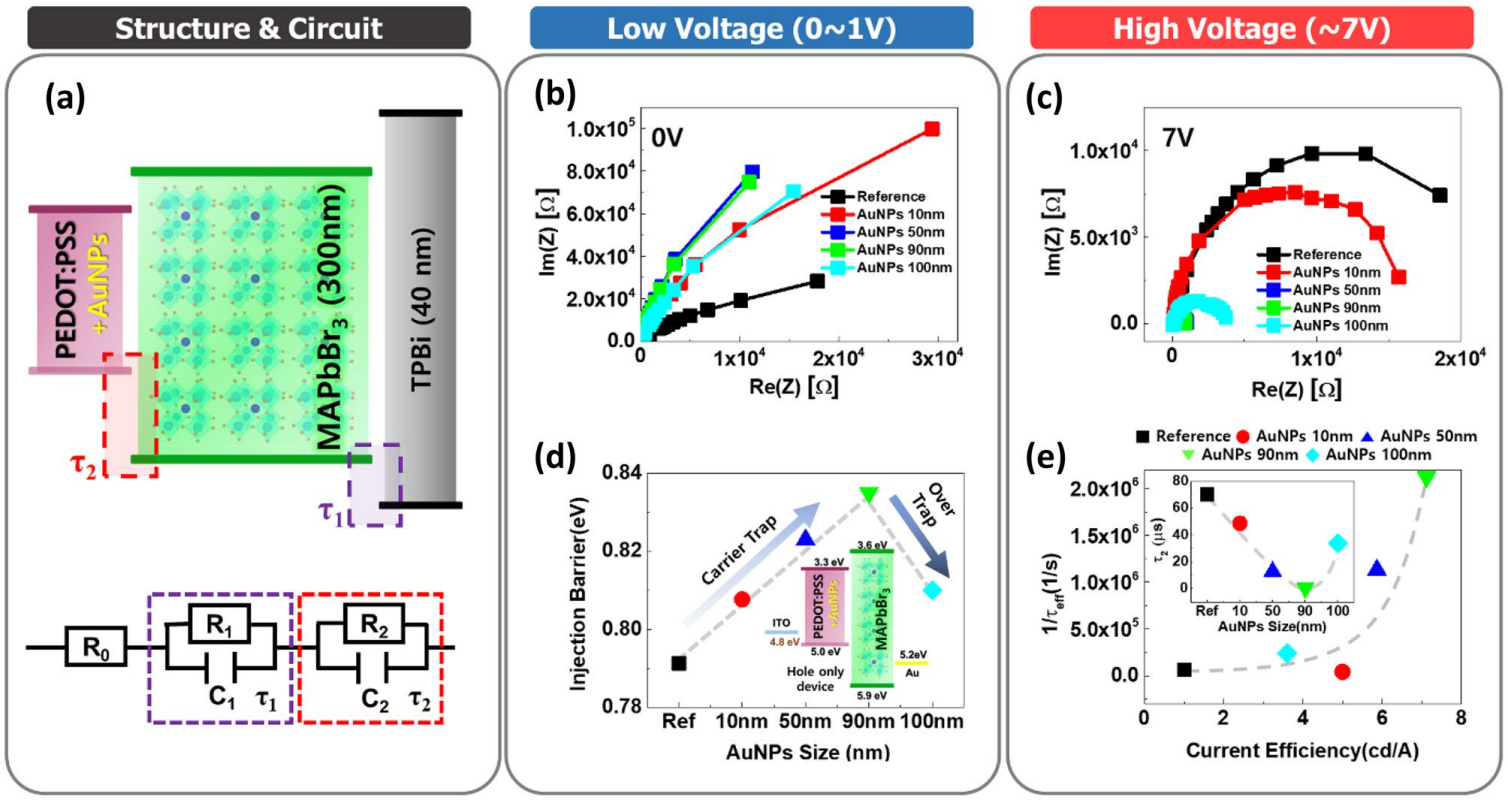
Electrical analysis based on the Cole–Cole plot. (**a**) Schematic of the band diagram and circuit modeling. (**b**, **c**) The Cole–Cole plot of AuNP PeLEDs in the condition of low and high voltage, respectively. (**d**) Injection barrier calculation result based on the hole-only-devices structure with the Richardson–Schottky equation e, 1/τ_eff_—current efficiency graph showing the optimal point of AuNP size. The inset is the τ_2_—AuNPs size, which shows the size-dependent trend.

**Figure 6:**
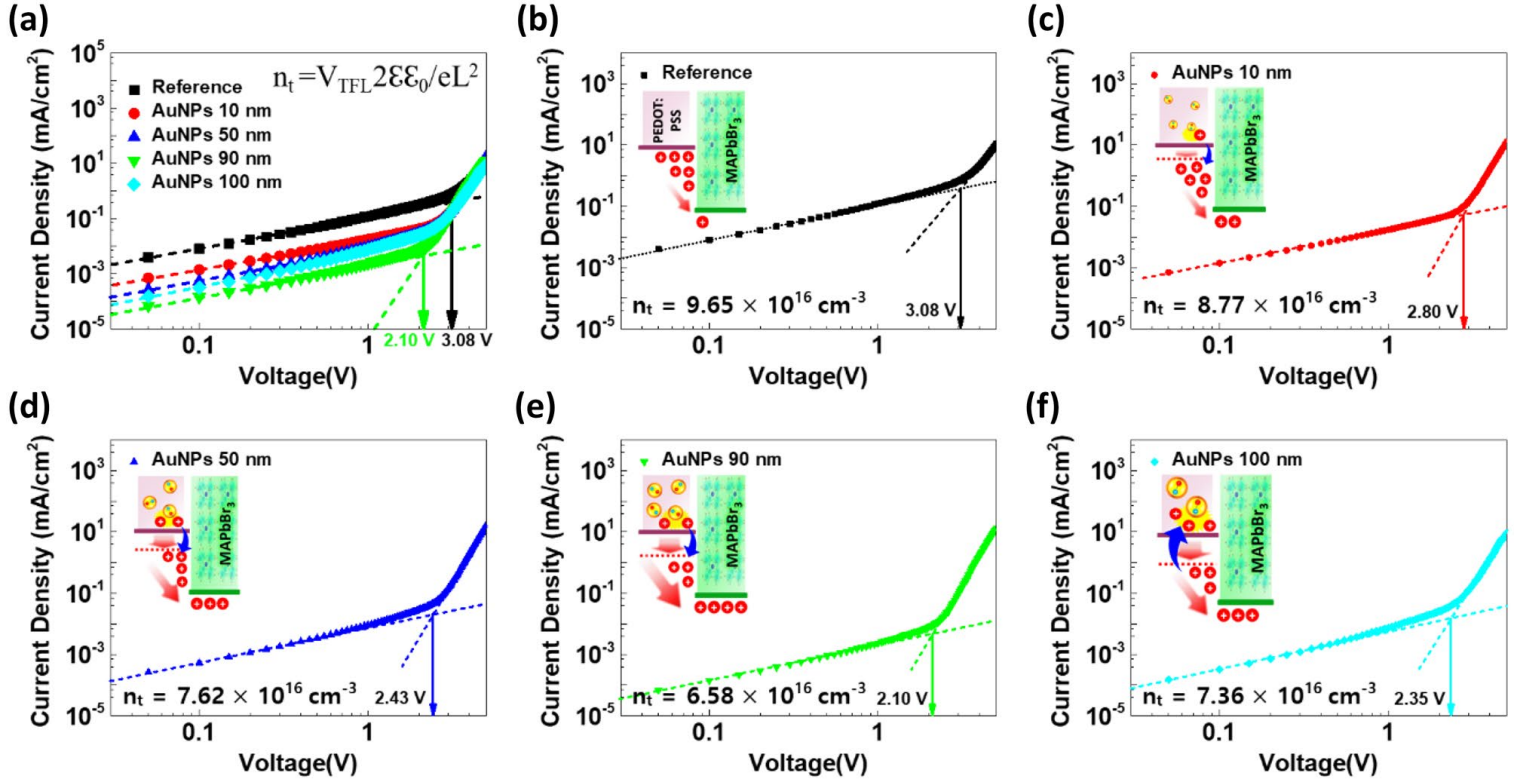
Trap-filled limit density analysis of AuNP-modified PEDOT:PSS. (**a**) J–V curve of the hole-only-device with and without AuNPs. (**b**–**f**) Current injection graphs of reference, 10, 50, 90, and 100 nm AuNPs with the schematic illustration of each condition.

We investigated the electrical properties of the PeLEDs in three regions: low voltage (0–1 V), intermediate voltage (2–3 V), and high voltage (7 V). PeLEDs can be modeled by an equivalent circuit with a series connection of parallel-connected resistive (R) and capacitive (C) components according to each device layer, as shown in Fig. 5a. The R and C of each layer are extracted from the real and imaginary impedance terms. R_0_, R_1_, R_2_ are the contact resistance for the ITO and LiF/Al contact, the interface between MAPbBr_3_/TPBi, and the interface between PEDOT:PSS/MAPbBr_3_, respectively. In this region, the negligible electric field (with 30 mV of the oscillation voltage) indicates that the increment in impedance is due to carrier trapping by AuNPs and PVP capping as passivation^34^. Compared with the reference device, the experimental group with AuNPs has higher impedance because AuNPs generate traps and make the injection barrier higher in the interface between PEDOT:PSS and MAPbBr_3_, whereas the device with 100 nm AuNPs showed reduced impedance due to the critically excessive trapped carriers by the large surface area of AuNPs (Fig. 5b). The *J–V* characteristics of the hole only devices (HODs) (ITO/PEDOT:PSS with or without AuNPs/MAPbBr_3_/Au, as shown in the inset of Fig. 5d), revealed that the devices containing AuNP-modified PEDOT:PSS layers exhibited a larger injection barrier than that of the pristine PEDOT:PSS-based device (Fig. 5d), considering only the lower voltage region (0–1 V). HODs were constructed as a double layer with perovskite because a short circuit occurs when only PEDOT:PSS is deposited. The hole injection barrier was increased by increasing the size of AuNPs from 10 to 90 nm owing to the increased carrier trapping, except in the case of 100-nm AuNPs because the injection barriers could also be affected by both carrier trapping/detrapping and the WF alignment (Table 3). PEDOT:PSS with 90 nm AuNPs showed the highest injection barrier. The injection barrier was measured according to the Richardson–Schottky equation that expresses the current density as an exponential function of the square root of the electric field (Fig. 6)^58,59^. Although the barrier from the PEDOT:PSS layer to the OIHP layer is induced by AuNPs, the overall energy barrier from the ITO to the OIHP layer is not always reduced owing to carrier trapping. By adding AuNPs to PEDOT:PSS, the current injection can be improved because of WF alignment but has a critical point at AuNPs 90 nm. In other words, carrier trapping is dominant until the size of the AuNPs is 90 nm (injection barrier increases), while carrier detrapping occurs for 100-nm AuNPs, which reduces the injection barrier in the lower voltage region (0–1 V).

**Table 3:** V_TFL_, n_t_, and injection barrier calculated from the hole-only-device and 1/τ_eff_ and 1/τ_2_ from the full device.

	HOD			Full device	
	V_TFL_ (V)	n_t_ (cm^−3^)	Injection barrier (eV)	1/τ_eff_ (1/s)	τ_2_ (μs)
Reference	3.08	9.65 × 10^16^	0.791	6.35 × 10^5^	70.13
AuNPs 10 nm	2.80	8.77 × 10^16^	0.807	4.11 × 10^5^	48.68
AuNPs 50 nm	2.43	7.62 × 10^16^	0.823	1.13 × 10^6^	12.74
AuNPs 90 nm	2.10	6.58 × 10^16^	0.835	2.12 × 10^6^	0.635
AuNPs 100 nm	2.35	7.36 × 10^16^	0.810	2.41 × 10^5^	33.90

In the high-voltage region, all the devices were sufficiently turned on at 5–7 V. In this bias region (Fig. 5c), the devices with AuNPs have lower impedance than those without AuNPs owing to the reduced charge accumulation by the WF alignment instead of carrier trapping, except in the case of 100-nm AuNPs (Table 3). At a low electric field, we can confirm only the trapped carriers at the AuNPs, not mainly the carrier accumulation and injection barrier, whereas, at a high electric field, we were able to confirm the total charge injection, including carrier trapping/detrapping at AuNPs by enormous carriers injected with high energy^25^. Since there are already enough holes to jump over the trap, holes are less affected by the traps owing to large carrier accumulation by the high electric field. Additionally, the small HOMO offset (between AuNP-modified PEDOT:PSS and MAPbBr_3_) facilitates hole transport because the WF of the modified PEDOT:PSS is related to the diameter of the AuNPs^60^. It is consistent with the results in Fig. 3a. Overall, we can conclude that the device efficiency and the effects of AuNPs in PEDOT:PSS are negatively boosted by carrier trapping at AuNPs in the low-voltage region, whereas they are positively boosted by the carrier detrapping in the high-voltage region and the band alignment due to WF modification. Therefore, the critical point for the above modeling was found when the size of the AuNPs was over 90 nm; however, carrier trapping was still observed for 100-nm AuNPs owing to the larger surface area.

The effective time delay (τ_eff_) is a key factor in determining the device performance and is calculated by multiplying the extracted circuit components (R∙C). According to Mathieson’s law, τ_eff_ is described by the harmonic average of time delays^61^. In the case of the conventional circuit model, a large τ_eff_ degrades the total current density, which means that the driving current of PeLEDs is suppressed by poor carrier transportation^61^. Compared with the reference device, τ_eff_ was reduced by increasing the diameter of AuNPs, except for 100-nm AuNPs, due to excessive trapped carriers. From the correlation between 1/τ_eff_ and CE (Fig. 5e and Table 3), it was found that the correlation between 1/τ_eff_ and current exponentially increased from 10 to 90 nm. These data confirm that the delay(τ) was the shortest, and the best efficiency was achieved for 90-nm AuNPs because the reduction in the delay time with the size of AuNPs in the 7 V condition improved hole injection and led to a greater possibility of exciton formation. To investigate the interface between PEDOT:PSS/MAPbBr_3_, τ_2_ was analyzed in detail (inset of Fig. 5e). A similar correlation was observed at τ_2_, and, although PEDOT:PSS without AuNPs exhibited a low injection barrier (without trapping by AuNPs), a large energy gap existed between the PEDOT:PSS and perovskite layers. Notably, for better hole injection, the optimum size of the AuNPs was found to be 90 nm. The delay time was also increased when the size of the AuNPs increased beyond 90 nm. Compared to the device without AuNPs, the results of the LT50 measurement in the device with AuNPs were improved because the defects caused by perovskite surface ion migration were reduced, and the non-radiative exciton quenching was also reduced.

To examine the electrical characteristics of the PeLEDs at an intermediate voltage (2–3 V), the trap-filled limit voltage (V_TFL_) was measured based on the HODs, as shown in Fig. 6a. In the intermediate region, the *J–V* curve showed an ohmic response at a voltage of approximately 2–3 V, while the current rapidly increased in the high-voltage region (over 3 V) exceeding the inflection point, indicating that the trap-sites were filled by the injected carriers^42^. Trap-density (n_t_) can be determined by V_TFL_ using the space charge limited current theory^62^. More information regarding the role of AuNPs can be obtained from charge trap-density, which is calculated using the equation V_TFL_ = en_t_L^2^/2εε_0_, where n_t_, e, ε, ε_0_, and L are the trap-state density, elementary charge, relative permittivity of the perovskite layer (25.5)^63^, vacuum permittivity, and the thickness of the perovskite layer (300 nm), respectively. n_t_ could be divided into two parts: carrier trapping/detrapping at AuNPs and carrier accumulation between PEDOT:PSS and the OIHP interface. In short, if the n_t_ value is low, the current injection is enhanced. In the case of a reference device without AuNPs (Fig. 6b and Table 3), the highest n_t_ (no carrier trapping without AuNPs, while carriers highly accumulated at the interface, and finally higher n_t_) was observed due to the mismatch of WF and carrier accumulation. However, n_t_ is reduced by the addition of AuNPs due to the increase in WF and the existence of charge carriers in AuNPs (slightly increased carrier trapping at AuNPs, less carrier accumulation at the interface, and finally lower n_t_), as shown in Fig. 6c–e and Table 3. For 100 nm AuNPs, n_t_ increased again, which indicates that an excessive number of carriers are trapped and negatively affect the current injection due to the critically expanded AuNP area, as suggested in Fig. 6f. In conclusion, using PVP-capped AuNPs, the current injection and device efficiency were enhanced owing to the plasmon effect, deeper WF of AuNP-modified PEDOT:PSS, and carrier trapping/detrapping mechanism.

The lack of AuNPs led to difficulty in hole injection due to the difference in HOMO energy levels between the hole injection layer and the emission layer^21,56^. Consequently, radiative recombination was decreased, and therefore, PeLED exhibited low efficiency. By contrast, the introduction of AuNPs into the PEDOT:PSS layer improved the charge injection by the WF alignment and carrier trapping/detrapping. Hence, hole injection became smoother, the recombination of charges increased, and exciton generation was enhanced, which led to a dramatic increase in the device efficiency of PeLED in a specific device structure with PEDOT:PSS and MAPbBr_3_ (Supplementary Fig. S9). A slightly deeper WF and smooth current injection were anticipated until the AuNPs reached 90 nm. However, as the surface area of the AuNPs became wider, the number of trapped carriers increased, and hole injection became more difficult. Various electrical analyses proved that PeLEDs with 90 nm AuNPs showed the highest efficiency, and those larger than 90 nm were not beneficial for PeLEDs because of the excessive carrier trapping by the larger surface area.

## Discussion

We demonstrated the dynamics of PeLEDs containing modified PEDOT:PSS layers with different sizes of AuNPs (10 50, 90, and 100 nm). By inserting AuNPs into the PEDOT:PSS layer, the WF was significantly improved, and the device performance of the PeLED was also elevated. CE was enhanced almost seven times from 1.05 to 6.99 cd A^−1^, and EQE was increased from 0.22 to 1.52%. This advancement was achieved owing to the better charge injection provided by AuNP-modified PEDOT:PSS and facilitated radiative recombination in the EML. TRPL results also confirmed that the increase in exciton lifetime and light out-coupling also increased because of the optical scattering properties caused by the plasmon effect. Seventeen times enhancement in a lifetime (LT50 from 88 to 1494 s) was achieved to avoid ion-diffusion/migration due to dipole formation by the PVP-capped AuNPs. Moreover, the results also confirmed that AuNPs larger than 100 nm are not suitable for PeLEDs, and the device efficiency and lifetime are reduced using AuNPs larger than 90 nm because the current injection is critically degraded by the excessive trapped carriers despite the enhanced optical property due to the plasmon effect. These findings indicate that the size and capping of MNPs play a vital role in avoiding defect production in PeLED devices. Lastly, the incorporation of MNPs into the HTL enhances the lifetime of the PeLED to a greater extent by electrical dipole formation. This proposed method can be conveniently applied to other perovskite materials such as FAPbBr_3_ and CsPbBr_3_ to solve the charge balance and stability problems of PeLEDs. This work will motivate us to use different nature of PVP-capped MNPs having different concentrations and sizes incorporated in PEDOT-based polymers with an in-depth perspective for future work.

## Methods

### Solution preparation

For the preparation of PEDOT:PSS layers modified by various sizes of AuNPs, different sizes of AuNPs (10, 50, 90, and 100 nm) were added in the PEDOT:PSS (Al4083 CLEVIOS) and isopropyl alcohol (IPA) under a 1.2:1:1 volume percentage^28^.

Although the commercial PVP-capped AuNPs were purchased from nanoComposix, Inc., a method for forming PVP capped AuNPs has been presented in several papers^64–66^. Generally, PVP was prepared with dissolution in water although the concentration of the PVP solution may be different and added it directly to the solution containing the citrate stabilized AuNPs. Methyl-ammonium-bromide (MABr) and lead bromide (PbBr_2_) were purchased from OSM. A 30 wt% MAPbBr_3_ solution was prepared by dissolving MABr and PbBr_2_ (mole ratio: 1.5:1) in DMF at 60 °C for 4 h^67^.

### PeLED device fabrication

The patterned ITO glass substrates were cleaned with acetone and IPA for 10 min in an ultrasonic bath at 40 kHz. Subsequently, the ITO substrates were treated with ultraviolet ozone (UVO, 187 nm) for 20 min. The sheet resistance of the ITO substrate was measured to be 15 Ω sq^−1^. Modified PEDOT:PSS was spin-coated onto the patterned ITO glass at 3000 rpm for 30 s and baked at 200 °C for 10 min in a nitrogen-filled glovebox. A precursor solution of MAPbBr_3_ was spin-coated using a one-step method. To form MAPbBr_3_ layers, MAPbBr_3_ solutions were spin-coated onto the PEDOT:PSS layers in the glovebox and then baked at 90 °C for 10 min. During MAPbBr_3_ spin-coating, nanocrystal pinning was conducted using a drop of chloroform:TPBi solution onto the spinning layers^8^. The fabrication of PeLEDs was completed by depositing TPBi, and the LiF/Al cathode was patterned using shadow masks by thermal evaporation with a base pressure of 2.0 × 10^–7^ Torr. The deposition rates were fixed at 1.0^1^, 0.2^1^, and 3.0 Å s^−1^, the thicknesses of TPBi, LiF, and Al were 40, 1, and 100 nm, respectively, as analyzed using crystal quartz and re-confirmed using a surface profiler. PeLEDs were encapsulated with a MgO moisture getter sheet before measurement in a glovebox with glass.

### PeLED device performance

The *J–V–L* and EL analyses for planar PeLEDs were conducted using a Keithley 2400 voltmeter and Minolta CS-2000 spectroradiometer.

### Surface morphology and roughness

The surface morphology of the deposited layers was analyzed using a UHR FE-SEM (S-5500, Hitachi) at the Korea Basic Science Institute (KBSI) Jeonju Center with an accelerating voltage of 10 kV. AFM was performed using an XE-7 (Park System, Korea) with a non-contact method.

### TOF–SIMS analysis

TOF–SIMS was performed using a Hybrid SIMS (ION-TOF GmbH, Münster, Germany) instrument at the KBSI Busan center. Bi_3_^+^ with a current 0.31 pA was used as a primary ion beam at an acceleration voltage of 30 keV, and the chemical images of the analyzed area (FoV, 200 μm × 200 μm) were recorded with 128 × 128 pixel resolution during data acquisition. The mass scale of the spectrum obtained from TOF–SIMS was calibrated using H^−^, C^−^, C_2_^−^, C_3_^−^, C_4_^−^, and C_8_^−^ in the negative mode. The sputter area was a square of 500 μm × 500 μm using Ar_n_^+^, where n =  ~ 1700 atoms as sputter argon gas cluster ion particles at 10 keV.

### UPS/XPS analysis

UPS spectra (AXIS-NOVA, Kratos. Inc.) were obtained with the samples biased at −15.0 V to clear the secondary cut-off. An energy resolution of 0.05 eV from the slope of the Fermi edge of a cleaned polycrystalline Au surface was utilized. XPS (AXIS-NOVA, Kratos. Inc.) with monochromatic Al-Kα (1486.6 eV) radiation as a photon source with a hemispherical analyzer was employed to investigate the chemical bonding nature of the species in the sample (base pressure = 1 × 10^−9^ Torr). High-resolution XPS patterns were obtained using an analysis area of 400 μm of the 40 eV pass energy with an energy step of 0.05 eV.

### XRD analysis

X-ray diffraction analysis was conducted using a Rigaku MiniFlex600 (Rigaku Corp., Tokyo, Japan) using Cu Kα radiation at 40 kV and 15 mA to identify the crystalline structures of the supports and catalysts in the 2θ range of 10°–80° at a scan speed of 10° min^−1^ with a step of 0.02°.

### CA analysis

The smart drop standard method using the FEMTO-BIOMED (South Korea) analyzer was adopted for the CA analysis. The system is equipped with a GEM single non-approximated algorithm numerical calculation based on the Bash forth-Adams equation.

### TRPL measurement

Time-resolved photoluminescence (TRPL) spectra were recorded using a femtosecond light source containing a home-built cavity-dumped Ti:sapphire laser with a 780-nm center wavelength (50 nm spectral width), 500 kHz repetition rate, and 30 fs pulse duration. An excitation beam was generated using a 200 µm thick LBO crystal (390 nm and 6.1 nJ). For TRPL measurement, the time-correlated single-photon counting (TCSPC) method was employed, which consisted of a commercial TCSPC board (SPC-130-EMN, Becker & Hickle Inc.). A single-photon avalanche photodiode (ID-100-50, IDQ Inc.) was used as the detector. A confocal setup using a parabolic mirror and rod mirror was also employed.

### Optical simulations

The full-wave numerical simulations were performed using the finite element method (COMSOL Multiphysics, RF module) to analyze the optical amplification by AuNPs, as shown in Fig. 4c,d. To calculate the radiation from the dipole in the PeLED, 30-nm-thick PEDOT:PSS and 300-nm-thick MAPbBr_3_ layers were sequentially placed on the ITO substrate, and AuNPs of different sizes were embedded in PEDOT:PSS. After that, the radiating dipole was placed near the AuNPs with varying positions relative to the AuNPs in the Y-axis, as shown in the inset of Fig. 4c. By considering the top emission of the PeLED, the radiation light energy normal to the surface of the MAPbBr_3_ layer was integrated. The optical amplification was calculated by normalizing the radiation light energy from the dipole with the AuNPs by that from the dipole without AuNPs. For all simulations, we assumed incoherent point dipole sources in the emissions.

## Supplementary Information

Supplementary Information.

## Data Availability

The data that support the plots within this paper and other findings of this study are available from the corresponding author upon reasonable request.
