# Author Correction: Improved device efficiency and lifetime of perovskite light-emitting diodes by size-controlled polyvinylpyrrolidone-capped gold nanoparticles with dipole formation

**DOI:** 10.1038/s41598-022-08896-5

**Published:** 2022-03-22

**Authors:** Chang Min Lee, Dong Hyun Choi, Amjad Islam, Dong Hyun Kim, Tae Wook Kim, Geon‑Woo Jeong, Hyun Woo Cho, Min Jae Park, Syed Hamad Ullah Shah, Hyung Ju Chae, Kyoung-Ho Kim, Muhammad Sujak, Jae Woo Lee, Donghyun Kim, Chul Hoon Kim, Hyun Jae Lee, Tae‑Sung Bae, Seung Min Yu, Jong Sung Jin, Yong‑Cheol Kang, Juyun Park, Myungkwan Song, Chang‑Su Kim, Sung Tae Shin, Seung Yoon Ryu

**Affiliations:** 1 Division of Display and Semiconductor Physics, Display Convergence, College of Science and Technology, Korea University Sejong Campus, 2511 Sejong‑ro, Sejong City, 30019 Republic of Korea; 2 Department of Applied Physics, Korea University Sejong Campus, 2511 Sejong‑ro, Sejong City, 30019 Republic of Korea; 3 E‑ICT‑Culture‑Sports Convergence Track, Korea University Sejong Campus, 2511 Sejong‑ro, Sejong City, 30019 Republic of Korea; 4 Department of Physics, Chungbuk National University, Cheongju, 28644 Republic of Korea; 5 Department of Electronics and Information Engineering, Korea University, Sejong, 30019 Korea; 6 Univ. Grenoble Alpes, Univ. Savoie Mont Blanc, CNRS, Grenoble INP, IMEP-LAHC, 38000 Grenoble, France; 7 Department of Advanced Materials Chemistry, College of Science and Technology, Korea University Sejong Campus, 2511 Sejong‑ro, Sejong City, 339‑770 Republic of Korea; 8 Jeonju Center, Korea Basic Science Institute (KBSI), 20, Geonji‑ro, Deokjin‑gu, Jeonju, Jeollabuk‑do 54907 Republic of Korea; 9 Busan Center, Korea Basic Science Institute (KBSI), Busan, 46742 Republic of Korea; 10 Department of Chemistry, Pukyong National University, 45 Yongso‑Ro, Nam‑gu, Busan, 48513 Republic of Korea; 11 Surface Technology Division, Advanced Nano‑Surface Department, Korea Institute of Materials Science (KIMS), Changwon, 51508 Republic of Korea

Correction to: *Scientific Reports* https://doi.org/10.1038/s41598-022-05935-z, published online 10 February 2022

The Acknowledgements section in the original version of this Article was incomplete.

“This research was supported by Basic Science Research Program through the National Research Foundation of Korea (NRF) funded by the Ministry of Education (NRF-2014R1A6A1030732, 2019R1F1A1060687, 2019R1C1C1006681, 2020R1A2B5B01001580). This work was supported by “Human Resources Program in Energy Technology” of the Korea Institute of Energy Technology Evaluation and Planning (KETEP), granted financial resource from the Ministry of Trade, Industry & Energy, Republic of Korea (No. 20204030200070). We also acknowledge support from the fundamental research program (PNK8100) of the Korea Institute of Materials Science (KIMS). Also, this work was supported by the Technology Innovation Program (20010804, Development of solution type polarizing materials and thin film circular polarizer for flexible OLED applications thickness below 30 μm transmittance above 41% and polarization efficiency above 98%) funded By the Ministry of Trade, Industry & Energy (MOTIE, Korea). This research was supported by the BK21 FOUR (Fostering Outstanding Universities for Research) funded by the Ministry of Education (MOE, Korea) and National Research Foundation of Korea (NRF).”

now reads:

“This research was supported by Basic Science Research Program through the National Research Foundation of Korea (NRF) funded by the Ministry of Education (NRF-2014R1A6A1030732, 2019R1F1A1060687, 2019R1C1C1006681, 2020R1A2B5B01001580). This work was supported by “Human Resources Program in Energy Technology” of the Korea Institute of Energy Technology Evaluation and Planning (KETEP), granted financial resource from the Ministry of Trade, Industry & Energy, Republic of Korea (No. 20204030200070). We also acknowledge support from the fundamental research program (PNK8100) of the Korea Institute of Materials Science (KIMS). Also, this work was supported by the Technology Innovation Program (20010804, Development of solution type polarizing materials and thin film circular polarizer for flexible OLED applications thickness below 30 μm transmittance above 41% and polarization efficiency above 98%) funded by the Ministry of Trade, Industry & Energy (MOTIE, Korea). This research was supported by the BK21 FOUR (Fostering Outstanding Universities for Research) funded by the Ministry of Education (MOE, Korea) and National Research Foundation of Korea (NRF). This research was supported by “IBET(IT/BT/ET) application technology using biosilica from freshwater diatom” of the Korea Environmental Industry & Technology Institute (KEITI) (No. 2021003270007)."

The original Article has been corrected.

