# Supplementary material for: Improved device efficiency and lifetime of perovskite light-emitting diodes by size-controlled polyvinylpyrrolidone-capped gold nanoparticles with dipole formation
Source: Sci Rep. 2022 Feb 10;12:2300. doi: 10.1038/s41598-022-05935-z (PMC8831638; doi:10.1038/s41598-022-05935-z)
Supplement: Supplementary file 1 — Supplementary Information. [file 41598_2022_5935_MOESM1_ESM.docx]

Supplementary Information

**Improved Device Efficiency and Lifetime of Perovskite Light-Emitting Diodes by Size-Controlled Polyvinylpyrrolidone-Capped Gold Nanoparticles with Dipole Formation**

Chang Min Lee^1,2,3,*^, Dong Hyun Choi^1,2,3,*^, Amjad Islam^1,2,3,*^, Dong Hyun Kim^1,2,3^, Tae Wook Kim^1,2,3^, Geon-Woo Jeong^1,2,3^, Hyun Woo Cho,^1,2,3^ Min Jae Park^1^, Syed Hamad Ullah Shah^1,2,3^, Hyung Ju Chae^1,2,3^, Kyoung-Ho Kim^4,^
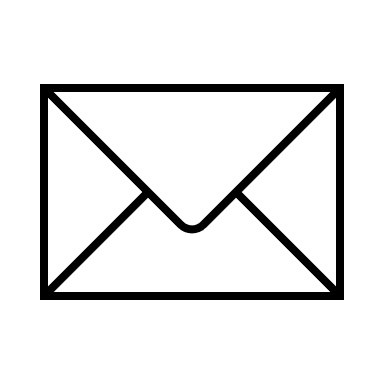
, Muhammad Sujak^4^, Jae Woo Lee^5^, Donghyun Kim^5,6^, Chul Hoon Kim^7^, Hyun Jae Lee^7^, Tae-Sung Bae^8^, Seung Min Yu^8^, Jong Sung Jin^9^, Yong-Cheol Kang^10^, Juyun Park^10^, Myungkwan Song^11^, Chang-Su Kim^11,^
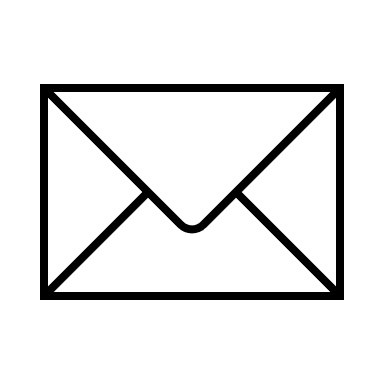
, Sung Tae Shin^1,2,3^ and Seung Yoon Ryu^1,2,3,^
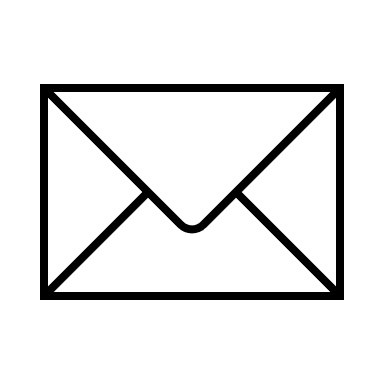


*^1^Division of Display and Semiconductor Physics, Display Convergence, College of Science and Technology, Korea University Sejong Campus, 2511 Sejong-ro, Sejong City 30019, Republic of Korea*

*^2^Department of Applied Physics, Korea University Sejong Campus, 2511 Sejong-ro, Sejong City 30019, Republic of Korea*

*^3^E-ICT–Culture-Sports Convergence Track, Korea University Sejong Campus, 2511 Sejong-ro, Sejong City 30019, Republic of Korea*

*^4^Department of Physics, Chungbuk National University, Cheongju 28644, Republic of Korea*

*^5^Department of Electronics and Information Engineering, Korea University, Sejong 30019, Korea*

*^6^Univ. Grenoble Alpes, Univ. Savoie Mont Blanc, CNRS, Grenoble INP, IMEP-LAHC, 38000 Grenoble, France*

*^7^Department of Advanced Materials Chemistry, College of Science and Technology, Korea University Sejong Campus 2511 Sejong-ro, Sejong City, 339-770, Republic of Korea*

*^8^Jeonju Center, Korea Basic Science Institute (KBSI), 20, Geonji-ro, Deokjin-gu, Jeonju, Jeollabuk-do, 54907, Republic of Korea*

*^9^Busan Center, Korea Basic Science Institute (KBSI), Busan, 46742, Republic of Korea*

*^10^Department of Chemistry, Pukyong National University, 45 Yongso-Ro, Nam-gu, Busan 48513, Republic of Korea*

*^11^Surface Technology Division, Korea Institute of Materials Science (KIMS) Changwon, 51508, Republic of Korea*

*These authors contributed equally to this work.


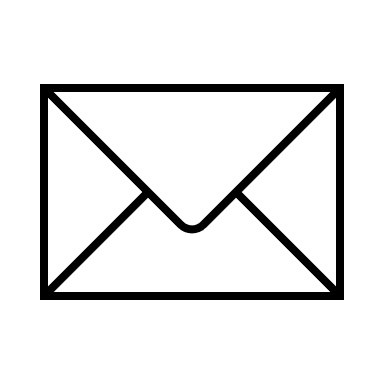
Corresponding author List

Prof. Dr. Seung Yoon Ryu^1, 2, 3,^
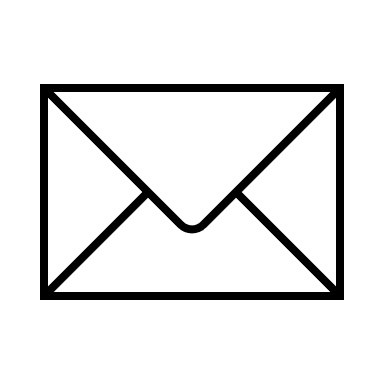


^1^Division of Display and Semiconductor Physics, College of Science and Technology, Korea University Sejong Campus 2511 Sejong-ro, Sejong City, 30019, Republic of Korea,

^2^Department of Applied Physics, Korea University Sejong Campus, 2511 Sejong-ro, Sejong City 30019, Republic of Korea

^3^E-ICT–Culture-Sports Convergence Track, Korea University Sejong Campus, 2511 Sejong-ro, Sejong City 30019, Republic of Korea

Tel) +82-44-860-1376, justie74@korea.ac.kr

Prof. Dr. Kyoung-Ho Kim^4,^
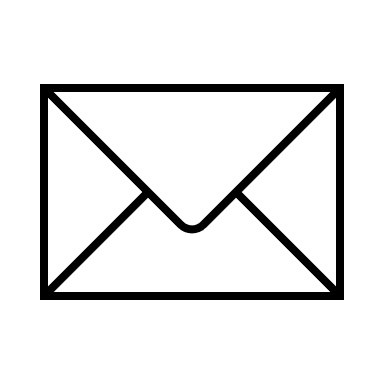


^4^Department of Physics, Chungbuk National University, Cheongju 28644, Republic of Korea

Tel) +82-43-261-2271, kyoungho@chungbuk.ac.kr

Dr. Chang-Su Kim^11,^
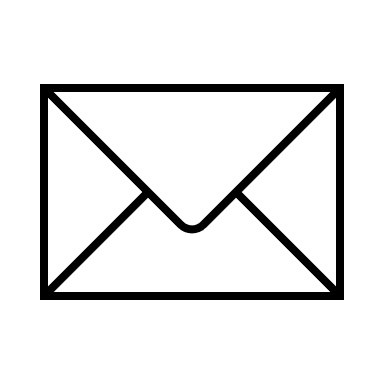
 ^11^Advanced Nano-Surface Department, Korea Institute of Materials Science (KIMS), Changwon 51508, Republic of Korea Tel)+82-55-280-3696, [cskim1025@kims.re.kr](mailto:justie74@korea.ac.kr)

**Keywords:** Perovskite Light-Emitting Diodes, Gold Nanoparticles, Plasmon Effect, Induced Dipole, Polyvinylpyrrolidone-Capping, Ion Diffusion/Migration, Far-Field Light Scattering**
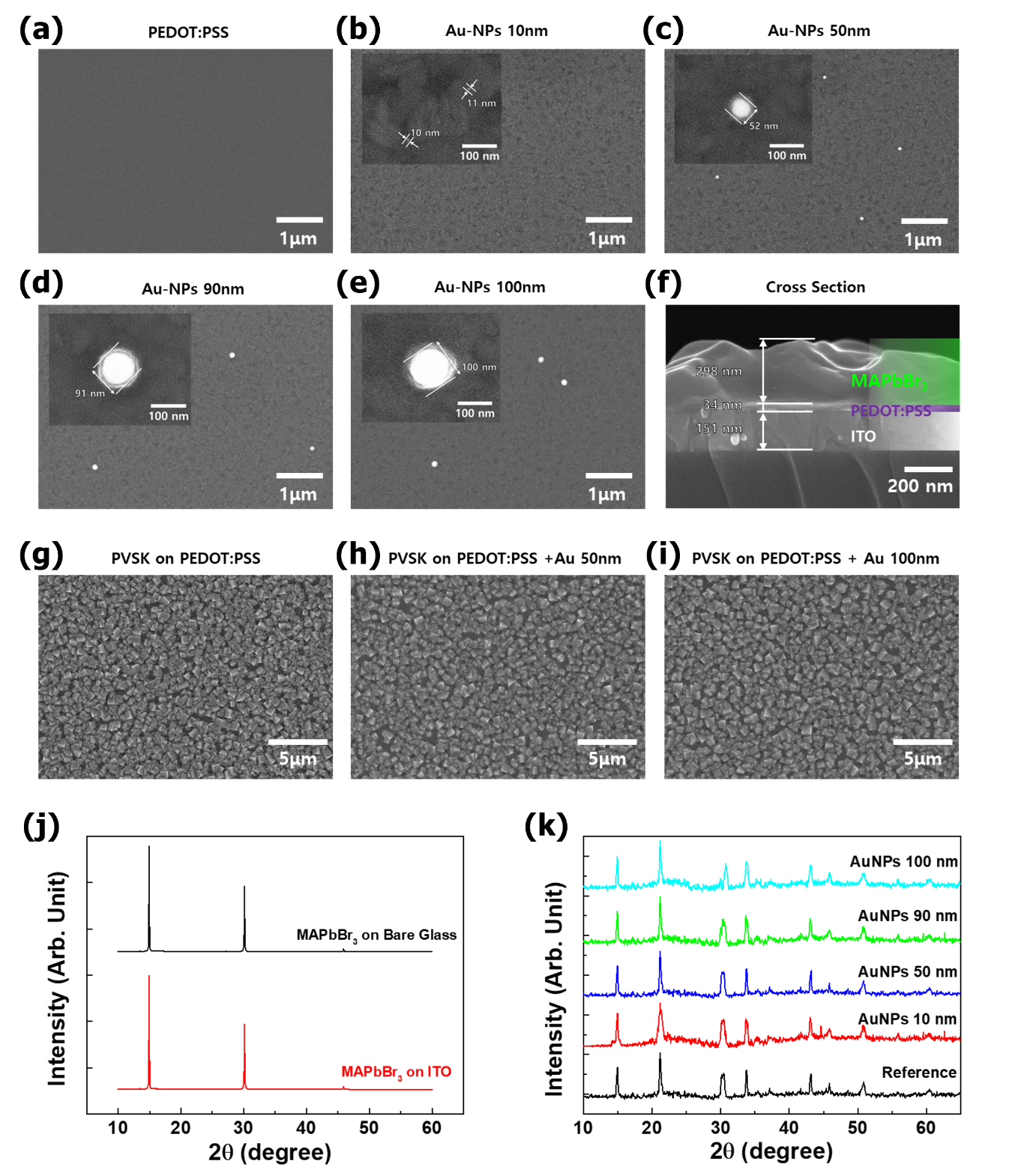
**

**Figure S1. UHR FE-SEM images and XRD analysis.** Surfaces of the **(a)** pristine and **(b)** 10 nm, **(c)** 50 nm, **(d)** 90 nm, and **(e)** 100 nm AuNP containing PEDOT:PSS on ITO, where the AuNPs were partially covered with 30 nm thick PEDOT:PSS. **(f)** Cross-sectional view of the MAPbBr_3_ on the pristine PEDOT:PSS. Surfaces of the PVSK on **(g)** pristine and **(h)** 50 nm and **(i)** 100 nm AuNP containing PEDOT:PSS. The XRD result of MAPbBr_3_ on **(j)** the bare glass and ITO glass and **(k)** AuNP-modified PEDOT:PSS (Reference, AuNPs 10 nm, 50 nm, 90 nm, and 100 nm) on ITO glass

We calculated the coverage densities of AuNP-modified PEDOT:PSS based on the equation below:

$Coverage Density= \frac{number of AuNPs \times area occupied by one particle}{Total area}$

The estimated coverage densities of Au-NPs in PEDOT:PSS with respect to different sizes (AuNPs 10 nm, 50 nm, 90 nm, and 100 nm) were 0.18 % ± 0.03 %, 1.18 % ± 0.26 %, 2.54 %± 0.85 %, and 2.36 % ± 1.03%, respectively. Although the coverage densities of the AuNPs were low, they played an important role in the optical and electrical properties of the devices.

**
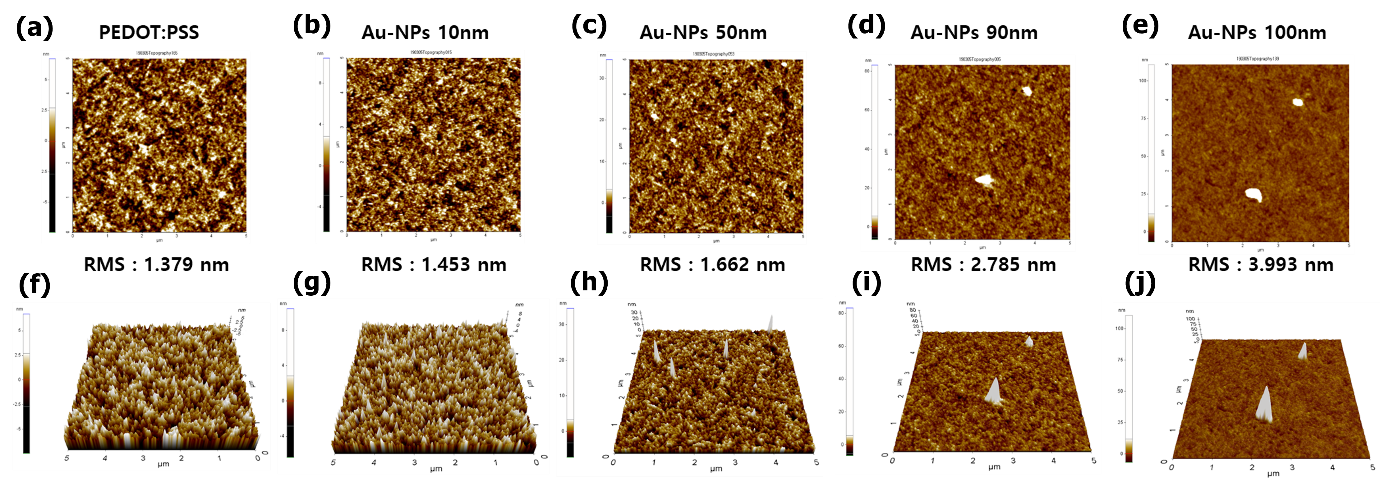
**

**Figure S2.** **AFM images for the surface morphology and roughness.** Surface of the **(a)** pristine and **(b)** 10 nm, **(c)** 50 nm, **(d)** 90 nm, and **(e)** 100 nm AuNPs containing PEDOT:PSS on ITO and **(f)**–**(j)** the corresponding 3D images.


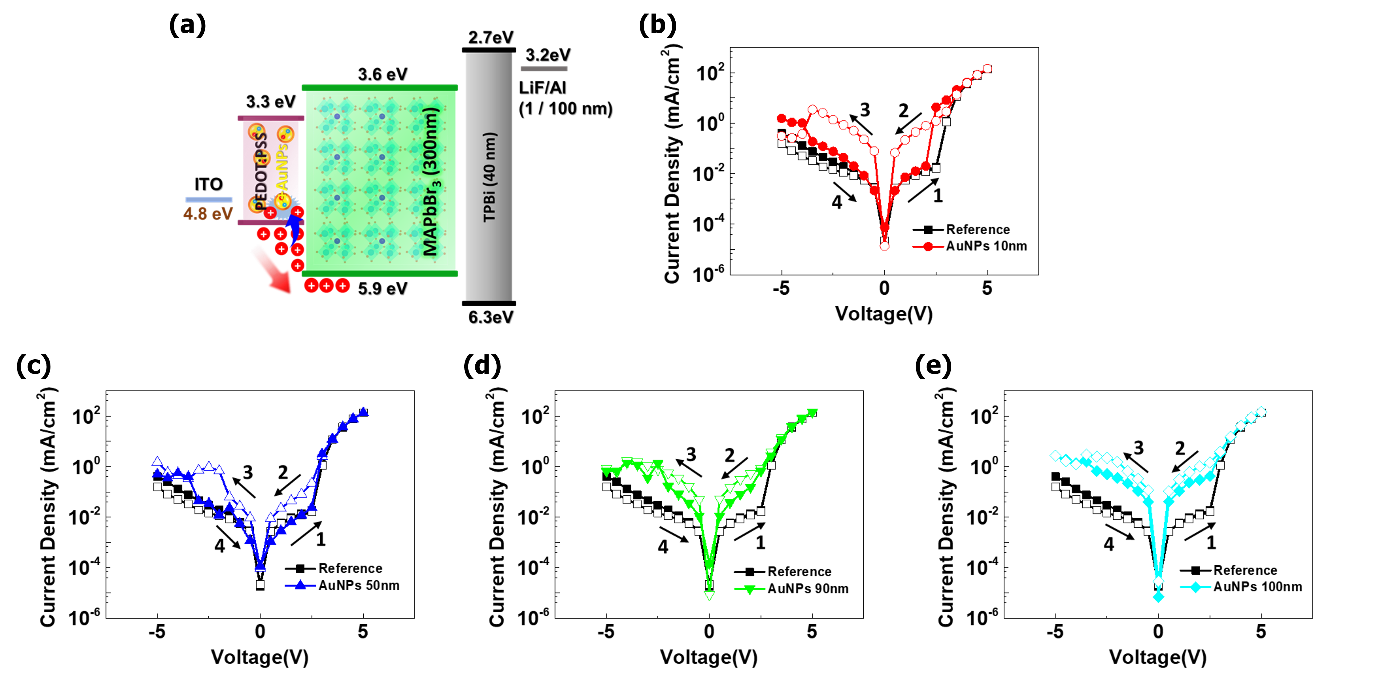


**Figure S3: IV cycle of the full device with and without AuNP-modified PEDOT:PSS** **(a)** Schematic illustration of the full device. **(b)**–**(e)** Current injection graph for different AuNPs.

The forward and reverse scans differ, which can explain the dipole formation in the PEDOT:PSS layer. Furthermore, the overall high current injection compared to the reference device is due to the deeper work function.

The injection barrier was measured according to the Richardson–Schottky equation that expresses the current density as an exponential function of the square root of the electric field.^1^ We incorporated the Richardson–Schottky equation as follows:

$$J_{RS}=A^{*}T^{2}exp\left[ \frac{ɸ_{B}-\beta_{RS}\sqrt{E}}{K_{B}T} \right]$$

where A is the Richardson constant, *T* is the temperature, K_B_ is the Boltzmann's constant, *E* is the electric field, *J*_RS_ is the current density, and ɸ_B_ is the zero-field hole injection barrier. $\beta_{RS}$ = $\sqrt{q/4\pi\varepsilon\varepsilon_{0}}$; here, q, ε, and ε_0_ represent the electronic charge, permittivity of free space, and in the medium, respectively.^2^

**
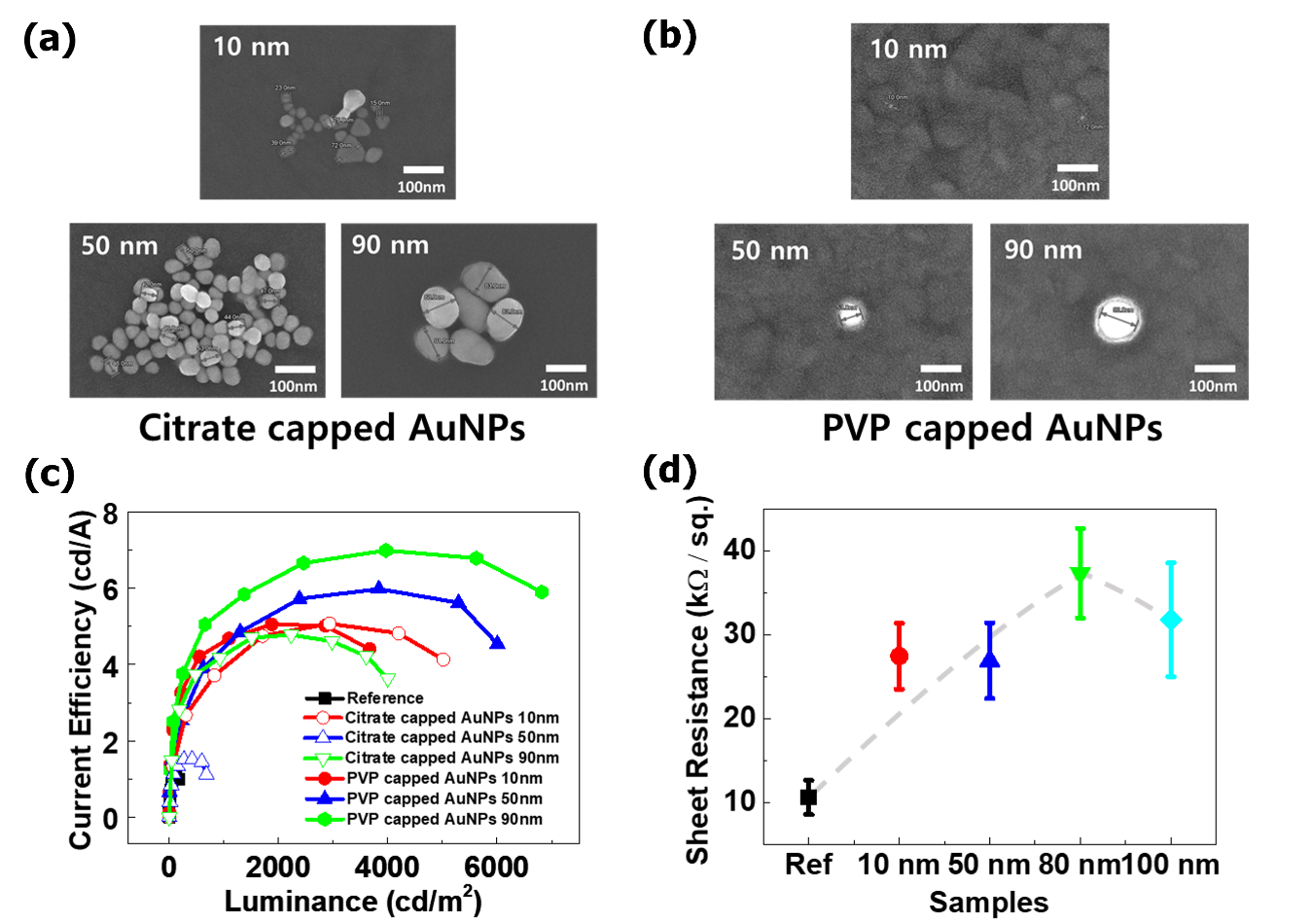
**

**Figure S4: UHR-FE SEM images of AuNP with different capping materials. (a)** Citrate and **(b)** PVP capped with 10, 50, and 90 nm AuNPs. **(c)** Current efficiency-luminance curves of the devices with AuNPs of different sizes and capping materials. **(d)** Sheet resistance of PVP-capped AuNPs

Inspired by our previous work related to the application of AuNPs in solar cells, citrate-capped AuNPs were used in PeLED devices.^3^ However, the conductivity/resistance of PEDOT:PSS with the citrate-capped AuNP film was increased/decreased by increasing the size of AuNPs due to the aggregation of AuNPs. In contrast, PVP-capped AuNPs showed a tendency to increase sheet resistance owing to the isolated AuNPs. Citrate-capped AuNPs exist in unstable regions of zeta-potential; thus, the actual size of AuNPs is increased due to aggregation, showing characteristics of AuNPs with a size of several hundred nanometers.^4^

As the PVP-capped AuNPs existed in the stable region of zeta-potential, they were well-dispersed and performed better than citrate-capped AuNPs. For the difference in sheet resistance between citrate and PVP-capped AuNPs, we infer that the carriers trapped by the induced electrical dipoles make the current path longer at the PVP-capped AuNPs, while carriers that go through aggregated citrate-capped AuNPs shorten the current path.


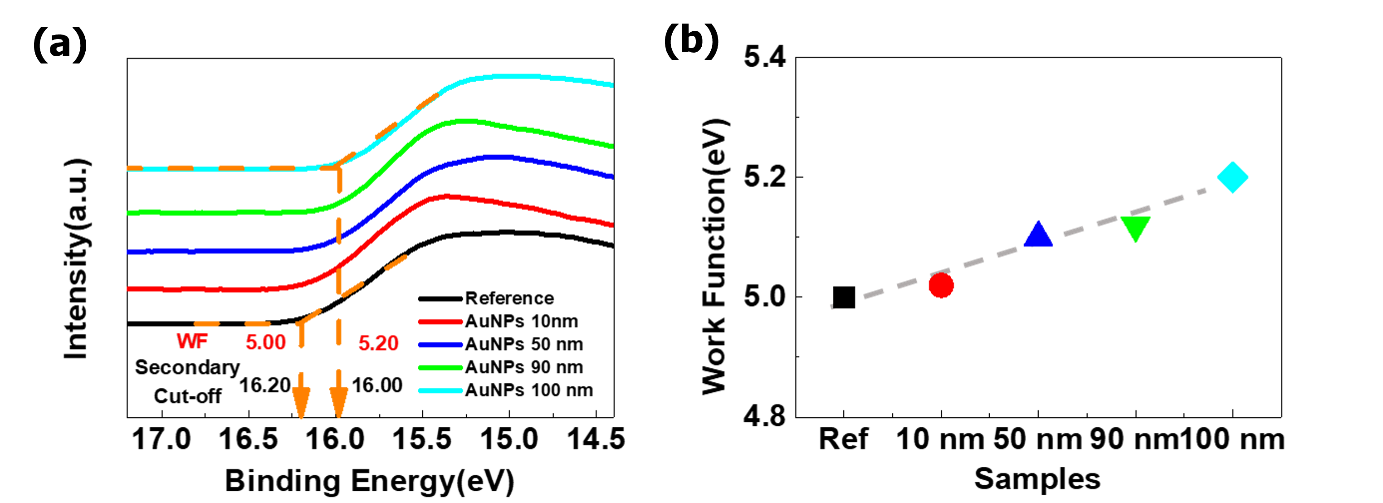


**Figure S5: The ultraviolet photoelectron spectroscopy (UPS) curves of MAPbBr_3_ on AuNP-modified PEDOT:PSS. (a)** The UPS spectra of different AuNP-modified PEDOT:PSS spin-coated onto ITO. **(b)** Graph of AuNP sizes and the value of the work function.

When metal NPs were added as an additive to a material such as a graphene oxide or self-assembled monolayer, their work function changed.^5, 6^ We observed similar effects on AuNP-modified PEDOT:PSS. Hence, smooth and better hole injection from ITO to OIHP was anticipated owing to the work function alignment by AuNP-modified PEDOT:PSS.

**
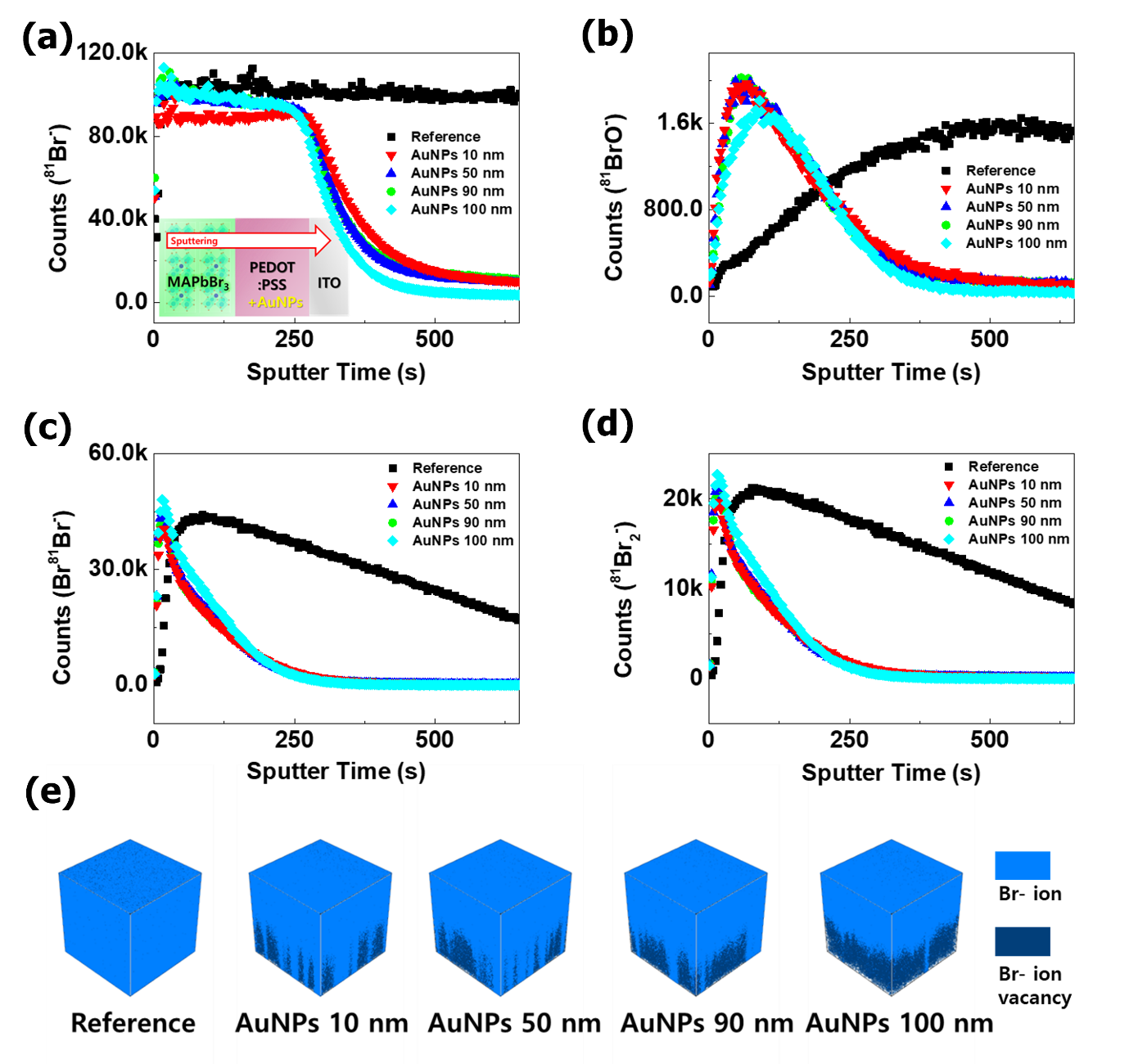
**

**Figure S6: Time-of-flight secondary ion mass spectrometry (TOF-SIMS) analysis of MAPbBr_3_ and ITO.** TOF-SIMS depth profiles of **(a)** ^81^Br^-^, **(b)** ^81^BrO^-^, **(c)** Br^81^Br^-^ and **(d)** ^81^Br_2_^-^. **(e)** The 3D distribution image of Br^-^ ions with and without AuNPs from the TOF-SIMS depth profiles.


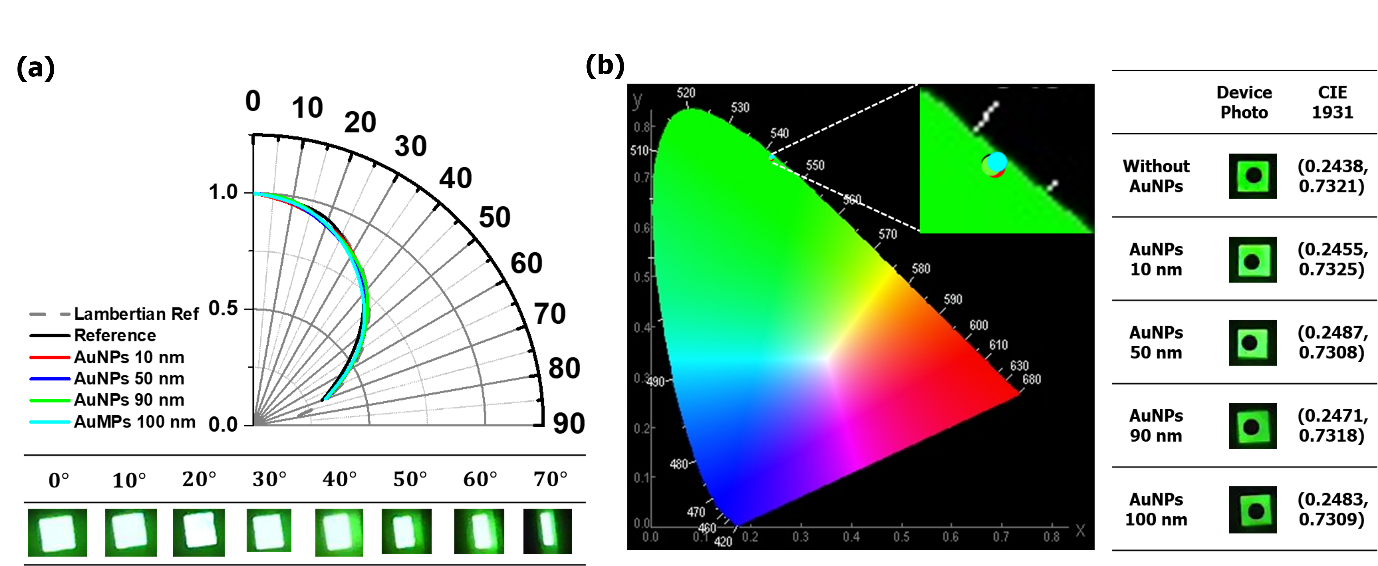


# Figure S7: Angle-dependence and the International Commission on Illumination (CIE-1931) coordinates of AuNPs modified PeLEDs (a) Angle distribution and (b) CIE 1931 color coordinate of PeLEDs

# The viewing angle measurement data of AuNPs-modified PeLEDs confirm that all devices followed the Lambertian trend (Figure S7a). This implies that light is randomly dispersed by AuNPs rather than directional scattering. An enlarged image in CIE 1931 coordinates (Figure S7b) reveals that AuNPs did not change the CIE coordinates.

**
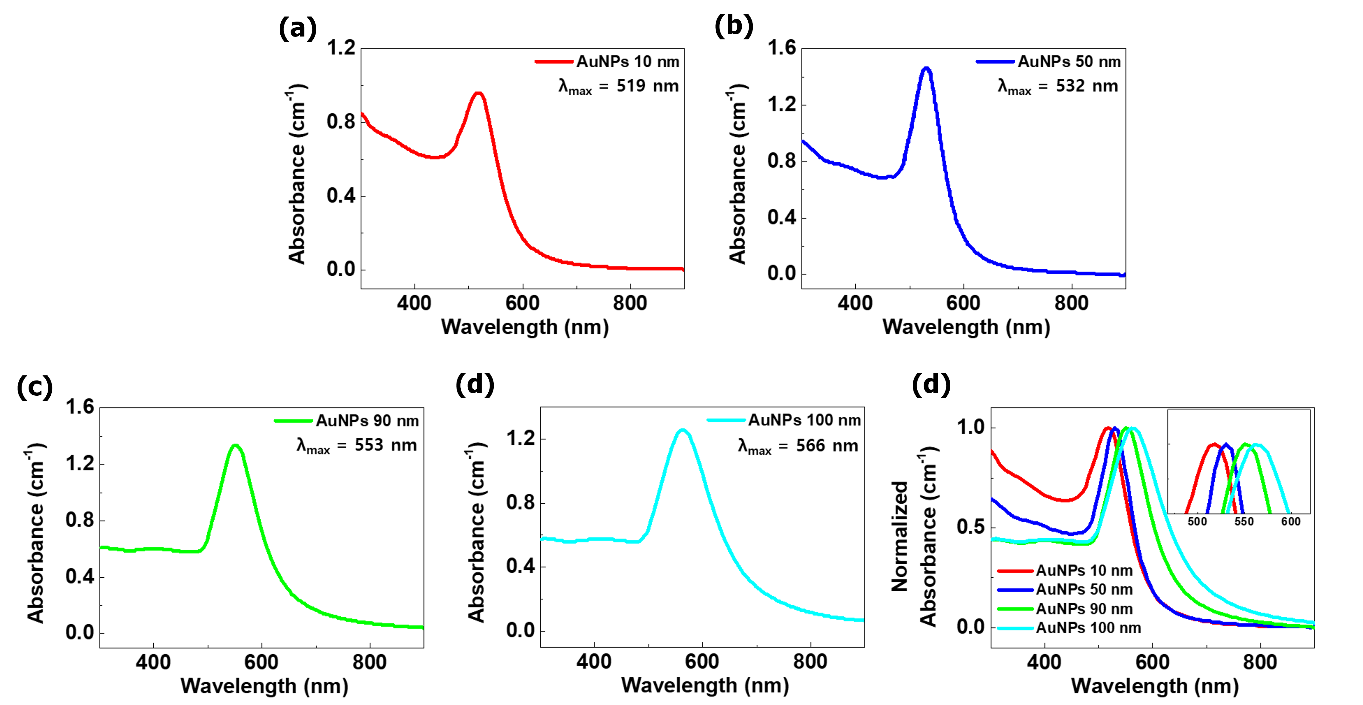
**

**Figure S8: UV-visible absorption spectra of AuNPs of sizes** **(a)** 10 nm, **(b)** 50 nm, **(c)** 90 nm, and **(d)** 100 nm, and **(e)** the normalized absorbance of each AuNPs

Different AuNPs from 10 nm to 100 nm exhibited absorption at different λ_max_ values, and the difference in wavelength was calculated by subtracting the value from the absorption of MAPbBr_3_ (used as EML), as shown in Figure S8. The peak emission wavelength of MAPbBr_3_ was observed at 538 nm. The difference in λ_max_ is approximately 20 nm for 10 nm AuNPs and about 28 nm for 100 nm AuNPs. In terms of absorption, 50 nm was the optimal size; however, considering the effects of optical scattering or electrical carrier trapping/detrapping, it is judged that 90 nm was the optimal size of AuNPs for optimal device performance.

| **Year** | **Previous Reports** | **HTL** | **Active Layer** | **ETL** | **CE** | **EQE** | **Lifetime**  **(LT50) (s)** |
| --- | --- | --- | --- | --- | --- | --- | --- |
| 2015 | Kim et al.^7^ | PEDOT:PSS  + PFI | MAPbBr_3_ | TPBi | 0.577 | 0.125 | - |
| 2015 | Hoye et al.^8^ | PEDOT:PSS | MAPbBr_3_ | F8 | 0.27 | Not reported | - |
| 2015 | Qin et al.^9^ | PEDOT:PSS | MAPbBr_3_ | TmPyPB | Not reported | 0.1 | - |
| 2016 | Shi et al.^10^ | PEDOT:PSS | MAPbBr_3_ | ZnO | Not reported | 0.06 | 240 |
| 2016 | Ling et al.^11^ | PEDOT:PSS | MAPbBr_3_ | PVK:PBD | Not reported | 0.48 | - |
| 2017 | Kim et al.^12^ | PEDOT:PSS | MAPbBr_3_ | PMMA | 1.12 | Not reported | 510 |
| 2019 | Lee et al.^13^ | PEDOT:PSS  /PVP | MAPbBr_3_ | TPBi | 9.32 | 2.15 | 780 |
| 2019 | Kim et al.^14^ | PEDOT:PSS | MAPbBr_3_ | TPBi+PFN | 1.52 |  | 99 |
| **2021** | **Our work** | PEDOT:PSS  +AuNPs | MAPbBr_3_ | **TPBi** | **7.12** | **1.5** | **1495** |


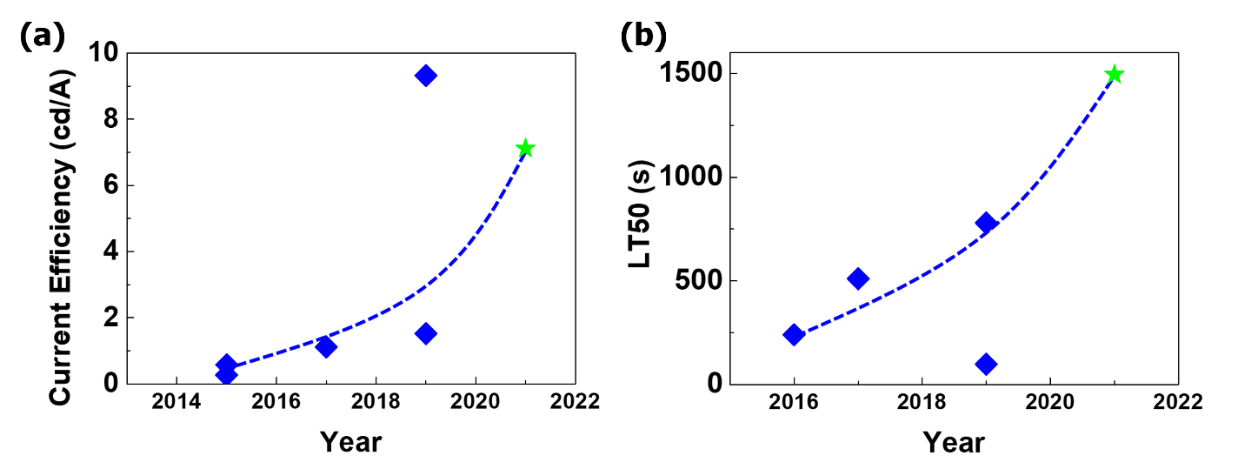


**Figure S9:** Comparison of this work with previous reports based on same active layer and HTL

Several attempts have been made to enhance the efficiency and lifetime of PeLED devices using conventional HTL (PEDOT:PSS) with OIHP (MAPbBr_3_). However, devices with a structure of ITO/PEDOT:PSS/MAPbBr_3_ with different ETLs did not show much improvement in efficiency. PEDOT:PSS is a well-known HTL and is extensively used in the commercial production of optoelectronic devices owing to its many advantages. Our strategy to incorporate the PVP-capped AuNPs into PEDOT:PSS has proved very effective in boosting the efficiency and lifetime of PeLED devices owing to the dipole formation to a large extent compared to the previously reported devices with ITO/PEDOT:PSS/MAPbBr_3_.

# References

1. Kim DH*, et al.* Improved charge balance in phosphorescent organic light-emitting diodes by different ultraviolet ozone treatments on indium tin oxide. *Organic Electronics* **61**, 343-350 (2018).

2. Brütting W, Berleb S, Mückl AG. Device physics of organic light-emitting diodes based on molecular materials. *Organic electronics* **2**, 1-36 (2001).

3. Shin J*, et al.* Harvesting near-and far-field plasmonic enhancements from large size gold nanoparticles for improved performance in organic bulk heterojunction solar cells. *Organic Electronics* **66**, 94-101 (2019).

4. Ngo VKT, Nguyen HPU, Huynh TP, Tran NNP, Lam QV, Huynh TD. Preparation of gold nanoparticles by microwave heating and application of spectroscopy to study conjugate of gold nanoparticles with antibody E. coli O157: H7. *Advances in Natural Sciences: Nanoscience and Nanotechnology* **6**, 035015 (2015).

5. Khoa NT, Kim SW, Yoo D-H, Kim EJ, Hahn SH. Size-dependent work function and catalytic performance of gold nanoparticles decorated graphene oxide sheets. *Applied Catalysis A: General* **469**, 159-164 (2014).

6. Schnippering M, Carrara M, Foelske A, Kötz R, Fermín DJ. Electronic properties of Ag nanoparticle arrays. A Kelvin probe and high resolution XPS study. *Physical Chemistry Chemical Physics* **9**, 725-730 (2007).

7. Kim YH*, et al.* Multicolored organic/inorganic hybrid perovskite light‐emitting diodes. *Advanced materials* **27**, 1248-1254 (2015).

8. Hoye RL*, et al.* Enhanced performance in fluorene‐free organometal halide perovskite light‐emitting diodes using tunable, low electron affinity oxide electron injectors. *Advanced Materials* **27**, 1414-1419 (2015).

9. Qin X, Dong H, Hu W. Green light-emitting diode from bromine based organic-inorganic halide perovskite. *Science China Materials* **58**, 186-191 (2015).

10. Shi Z-F*, et al.* High-performance planar green light-emitting diodes based on a PEDOT: PSS/CH 3 NH 3 PbBr 3/ZnO sandwich structure. *Nanoscale* **8**, 10035-10042 (2016).

11. Tan Z-K*, et al.* Bright light-emitting diodes based on organometal halide perovskite. *Nature nanotechnology* **9**, 687-692 (2014).

12. Kim YC, Porte Y, Baek S-D, Cho SR, Myoung J-M. High-Performance Green Light-Emitting Diodes Based on MAPbBr3–Polymer Composite Films Prepared by Gas-Assisted Crystallization. *ACS applied materials & interfaces* **9**, 44106-44113 (2017).

13. Lee H, Ko D, Lee C. Direct evidence of ion-migration-induced degradation of ultrabright perovskite light-emitting diodes. *ACS applied materials & interfaces* **11**, 11667-11673 (2019).

14. Kim T, Kim J-H, Triambulo RE, Han H, Park C, Park J-W. Improving the Stability of Organic–Inorganic Hybrid Perovskite Light‐Emitting Diodes Using Doped Electron Transport Materials. *physica status solidi (a)* **216**, 1900426 (2019).
